# The magic, memory, and curiosity fMRI dataset of people viewing magic tricks

**DOI:** 10.1038/s41597-024-03675-5

**Published:** 2024-10-01

**Authors:** Stefanie Meliss, Cristina Pascua-Martin, Jeremy I. Skipper, Kou Murayama

**Affiliations:** 1 School of Psychology and Clinical Language Sciences, University of Reading, Reading, UK; 2 Experimental Psychology, University College London, London, UK; 3 Hector Research Institute of Education Sciences and Psychology, University of Tübingen, Tübingen, Germany; 4 Research Institute, Kochi University of Technology, Kochi, Japan

**Keywords:** Long-term memory, Reward, Motivation

## Abstract

Videos of magic tricks offer lots of opportunities to study the human mind. They violate the expectations of the viewer, causing prediction errors, misdirect attention, and elicit epistemic emotions. Herein we describe and share the Magic, Memory, and Curiosity (MMC) Dataset where 50 participants watched 36 magic tricks filmed and edited specifically for functional magnetic imaging (fMRI) experiments. The MMC Dataset includes a contextual incentive manipulation, curiosity ratings for the magic tricks, and incidental memory performance tested a week later. We additionally measured individual differences in working memory and constructs relevant to motivated learning. fMRI data were acquired before, during, and after learning. We show that both behavioural and fMRI data are of high quality, as indicated by basic validation analysis, i.e., variance decomposition as well as intersubject correlation and seed-based functional connectivity, respectively. The richness and complexity of the MMC Dataset will allow researchers to explore dynamic cognitive and motivational processes from various angles during task and rest.

## Background & Summary

When a magician performs a magic trick, it is possible that for a moment, the boundaries between fantasy and reality can become blurry to the viewer because something logically impossible is shown. Magic tricks violate causal relationships and thereby expectations and predictions by producing an apparently implausible effect (like an object vanishing) using methods (how the trick works, e.g., by misdirection or illusion) that are unknown to the viewer, thereby producing a sense of wonder^1^. Because of such attributes, videos of magic tricks lend themselves well to studying the human mind scientifically (see^2^ for a review). For instance, magic tricks can be used to study attention^3^ because magic tricks often rely on attentional misdirection. On a broader level, because magic tricks violate expectations, they can be used to study the processing of prediction errors, the violation of causal relationships, and associated surprise^4,5^.

Magic tricks can also be used to elicit epistemic emotions^6^ (i.e., emotions crucial for learning and generating knowledge^7,8^) like curiosity – the intrinsic desire to know even in the absence of any instrumental value of the information. Studies in humans have found that curiosity leads to information seeking^9–13^ and enhances memory encoding and learning^14,15^. Evidence from functional magnetic resonance imaging (fMRI) studies suggests that higher feelings of curiosity are associated with increased activity in key structures known as the reward network^16–19^. Likewise, curiosity-dependent memory enhancements are supported by anticipatory activity in the hippocampus and dopaminergic midbrain^16,20,21^. These results led researchers to hypothesise that curiosity-driven behaviour can be understood as a type of reward-learning – people seek and encode the information based on its intrinsic rewarding value^10,22,23^.

**Fig. 1:**
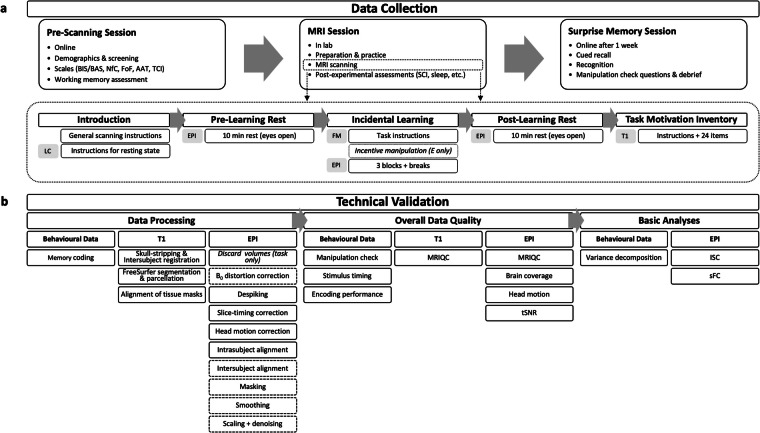
Illustration Summarising the Procedures used During Data Collection (top) and Technical Validation (bottom). In both, italic font indicates that certain procedures were used for a subset of participants/datasets. (**a**) The data collection involved three sessions: a pre-scanning session, a magnetic resonance imaging (MRI) session, and a surprise memory test. The upper part of the figure highlights which assessments were carried out during each session, whereas the lower part (dotted line) gives more details on the MRI scanning. The white rectangles summarise what was presented on the screen and the grey rectangles indicate which sequences were run simultaneously. (**b**) For the technical validation, data from different sources (behavioural data, functional data (EPI) and anatomical data (T1)) were preprocessed as indicated. Regarding the preprocessing of EPI data, solid lines indicate steps that were carried out in the minimal and full preprocessing pipeline, while dashed lines indicate that the steps were part of the full preprocessing pipeline but not the minimal preprocessing pipeline. To determine overall quality of imaging data, raw (MRIQC) or minimally preprocessed (brain coverage, head motion, tSNR) data were used, whereas fully preprocessed functional data were used for basic validation analysis (ISC, sFC). Abbreviations: BIS/BAS = Behavioural Inhibition/Activation Scale, NfC = Need for Cognition, FoF = Fear of Failure, AAT = Approach & Avoidance Temperament, TCI = Trait Curiosity Inventory, SCI = State Curiosity Inventory, E = experimental group, LC = localizer, EPI = echo-planar imaging, FM = field map, T1 = T1-weighted (anatomical) scan, MRIQC = software used to determine overall data quality, tSNR = temporal signal-to-noise ratio, ISC = intersubject correlation, sFC = seed-based functional connectivity.

Herein we describe, validate, and share the Magic, Memory, and Curiosity (MMC) Dataset (summarised in Fig. 1). It consists of behavioural and MRI data of 50 participants viewing 36 magic tricks performed by professional and semi-professional magicians. We also tested their incidental memory for the magic tricks one week later. The MMC Dataset is of particular importance in two respects. First, this is the first openly available fMRI dataset of magic trick viewing. Although there are a few fMRI studies using magic tricks, these studies did not use magic trick stimuli from a validated stimulus database, were limited in the number of magic trick videos presented, and/or participants included^4,5,18^. The MMC Dataset used a collection of well-validated magic trick video clips which we created specifically for fMRI experiments^6^. Second, this is the first openly available fMRI dataset that allows us to examine curiosity elicited by dynamic and complex stimuli. To induce curiosity inside the MRI scanner, previous fMRI studies on curiosity have predominantly relied on trivia questions promoting the awareness of a gap in their own knowledge^16,17,24^ or arbitrary lottery observing tasks during which participants can choose to reveal non-instrumental information^12,13^. While valuable, these tasks do not fully resemble curiosity as it manifests in the real-world, because paradigms are sparse, static, and discrete in a manner that misses the fundamental aspect of curiosity in our daily life — curiosity is normally triggered by complex and dynamic stimuli in the environment^22^.

fMRI data were acquired not only during task but also in rest periods before and after learning. We also manipulated an incentive context (i.e., whether participants anticipated monetary rewards or not), as well as measured participants’ subjective feelings of curiosity as a time-varying variable. This design provides us with additional richness and complexity^6^ to the data. As magic trick videos are all freely available with various normative ratings (e.g., surprise, confidence), researchers can have access to visual information as well as normative ratings, and they can even collect new variables of interest with online experiments. This allows researchers to study the neural mechanisms underlying various features of interests (e.g., curiosity, learning, incentives, surprise, prediction errors, or misdirection by the magician) with a plethora of possible analysis approaches and automatic annotation methods^25,26^. Indeed, the MMC Dataset can lend itself to let researchers compare the effects of analysing the data with different approaches (e.g., a standard General Linear Model (GLM) or the intersubject synchronisation framework^27,28^. The MMC Dataset also includes various individual differences variables (e.g., working memory, fear of failure, need for cognition), and the intersubject synchronisation framework used here to demonstrate data quality can further be extended to study such individual differences^29,30^. Furthermore, the availability of both pre- and post-learning phase resting scan data also makes it possible for researchers to examine how these complex brain dynamics and their individual differences during the learning session contribute to the change in post-learning resting-state brain activation. This can advance our understanding of how adaptive consolidation can support flexible, goal-relevant behaviours^31^. Thus, the MMC Dataset delivers substantial value for a broad neuroscientific community.

## Methods

### Participants and design

Due to a lack of previous fMRI studies investigating differential effects of incentives and curiosity on incidental encoding, sample size considerations were based on previous behavioural studies^32^, sample size recommendations to test for reliable between-group similarities and differences in neural responses to naturalistic stimuli^33,34^, and the results of behavioural pilot experiments with the same paradigm^30,35^. The *a priori* defined intended sample size was in total 50 participants (i.e., *N* = 25 in each the control and experimental group). This sample size is larger than what is typically found in standard fMRI experiments (including fMRI experiments on the topic of curiosity), deemed to have sufficient power to detect various within-person task-related activations (e.g., curiosity ratings). Please see the Usage Notes for further discussions on the issue of statistical power in the MMC Dataset.

To reach this number, leaflets were distributed on the university campus as well as in cafes and leisure centres. In total, we were approached by 110 interested participants via email who were then screened for MRI safety (e.g., no pacemaker or other active implants) and inclusion criteria. The latter required that participants be right-handed, fluent in English, aged between 18 and 45, healthy, not suffering from any chronic illness, psychiatric disorders or cognitive impairments, and not taking any psychoactive drugs. Moreover, participants needed to have normal hearing and normal or corrected vision using contact lenses. For females, it was additionally necessary that they were not nursing, pregnant, or intending to become pregnant.

In total, 64 participants met the inclusion criteria and consented to take part in the study. They were assigned ascending participant identification numbers in the order in which they contacted the research team (IDs, i.e., 1, 2, 3, etc.) that were assigned to the groups in interleaved manner: odd IDs were assigned to the control group (C), whereas even IDs were assigned to the experimental group (E). However, during the pilot phase of data collection (i.e., the first six participants), the first three participants were assigned to the control group whereas the remaining three participants were assigned to the experimental group. They were scheduled for all three sessions of data collection: (1) a pre-scanning online session, (2) an MRI session in the lab, and (3) an online surprise memory test a week after scanning. Four participants (2 in C, 2 in E) dropped out after consenting, but before completing the pre-scanning online assessment and another three participants (2 in C, 1 in E) completed the first session, but did not show up for the MRI scanning session in the lab. Another two participants (1 in C and 1 in E) withdrew their consent during the MRI session (one due to feeling claustrophobic and one due to a blocked nose). Additionally, five participants (2 C and 3 E) were excluded after acquisition but before data analysis due to technical issues during the scan. Any assigned IDs of dropped-out participants were reassigned until the intended sample size was met.

The final sample consisted of 50 participants (36 females, range of age 18–37 years, *M* = 25.32, *SD* = 5.19), most of them being research-naïve as no recruitment platform was used. Table 1 provides a summary of participant demographics for each group. The groups did not differ in demographics (all *p* ≥ .103).

**Table 1:** Description of Participants Means (Standard Deviation) in Each Group.

	Control Group	Experimental Group	Statistics group comparison
Age	26.52 (5.46)	24.12 (4.7)	*t*(46.96) = 1.665, *p* = .103
Sex assigned at birth (% female)	68	76	χ2 (1) = 0.099, *p* = .753
Ethnicity (% BAME)	32	24	χ^2 ^(1) = 0.099, *p* = .753
Years of Education	16.12 (2.62)	15.92 (2.04)	*t*(45.282) = 0.301, *p* = .765
Corsi span	5.56 (2.36)	5.8 (1.19)	*t*(35.43) = −0.453, *p* = .653
n-back (% accuracy)	73.83 (20.01)	65.84 (23.17)	*t*(47.007) = 1.306, *p* = .198
Experience with magic tricks	1.56 (0.87)	1.44 (0.77)	*t*(47.276) = 0.517, *p* = .608

All participants were right-handed and fluent in English. Sex is expressed as percentage female. Ethnic background is expressed as percentage Black, Asian, and Minority Ethnic (BAME). Statistics of group comparison report Two-Sample Welch *t*-test and χ^2^ (Chi-Square) test for continuous data (age, years of education, Corsi span, n-back accuracy, and experience with magic tricks) and categorical (sex and percentage BAME), respectively.

This study used a between-subject design manipulating whether performance in a pseudo task was incentivised: Participants in the experimental group were instructed that they could earn a performance-dependent additional bonus of £0.80 for each correct answer in the 36 trials of the magic trick watching task (see below). Participants in the control group did not receive such an instruction.

Participants were compensated with £30 for their participation in the study and all participants – regardless of the group they were assigned to – received an additional bonus payment of £7.20. This reflects chance level performance in the incentive-manipulated, four-alternative forced-choice pseudo task. The pseudo task was designed as part of the cover story to keep participants engaged and ensure they were paying attention; however, there were no correct or wrong answers. Hence, out of courtesy, all participants received the same bonus payment after the debrief at the end of the study.

The study design was approved by the University Research Ethics Committee (UREC) of the University of Reading (UREC 18/18). Participants provided informed written consent to participate in the study and to share their data in anonymised form. The study protocol was not pre-registered.

### Material

Magic trick stimuli were obtained from the Magic Curiosity Arousing Tricks (MagicCATs) stimulus set^6^ containing 166 short magic trick video clips. We selected 36 to use as stimuli in the experiment ensuring that they (a) had a duration between 20 and 60 s, (b) included a range of different materials and features so that they were distinguishable when used in a cued recall paradigm and (c) elicited curiosity to varying degrees based on the ratings reported in the database^6^. All videos in the database are muted on purpose to reduce verbal interferences. However, due to the non-verbal nature, the magic tricks are still understandable. Magic tricks relying on subtitles were excluded. The number of stimuli was equal to previous fMRI studies using magic tricks from the same database^18^. The final selection of stimuli was edited using Adobe^®^ Premiere Pro CC^®^ (2015) software to achieve a similar dark background and viewing focus. Where necessary, additional editing was performed, e.g., removing subtitles. The faces of the magicians were hidden as much as possible.

Furthermore, we created a mock video for each magic trick separately to capture transient, non-specific activity at stimulus onset^36^. For this, the first frame of the magic trick was used as a still image and presented for the duration of six seconds during which it was overlaid with a black video (opacity 99.7%) including a viewing focus that gradually expanded and smoothed to match the viewing focus used in each magic trick file. Mock video and magic tricks were combined into one video (range of duration in seconds 26.6–58.64, *M* = 38.53, *SD* = 8.63) and saved at a size of 1280 × 720 pixels in mp4. Additionally, we selected two more magic tricks to be used during the practice trials according to the same criteria and edited them as described above. A description of each magic trick used can be found in the Supplementary Information.

We rated each magic trick with respect to its moment(s) of surprise, i.e., the moment(s) of violation of expectations and then selected a frame for each magic trick from before the (first) moment of surprise that was distinctive enough to cue the respective magic trick without revealing the trick entirely. This frame was then used as a cue image (size 1920 × 1080 pixels) in the memory task. The timestamps of the moment(s) of surprise and cue image can be found in Supplementary Information. All final stimuli are available upon request. The request procedure is outlined in the Open Science Framework repository associated with the MagicCATs stimulus set (https://osf.io/ad6uc/).

### Tasks and measurements

#### Magic trick watching task

**Fig. 2:**
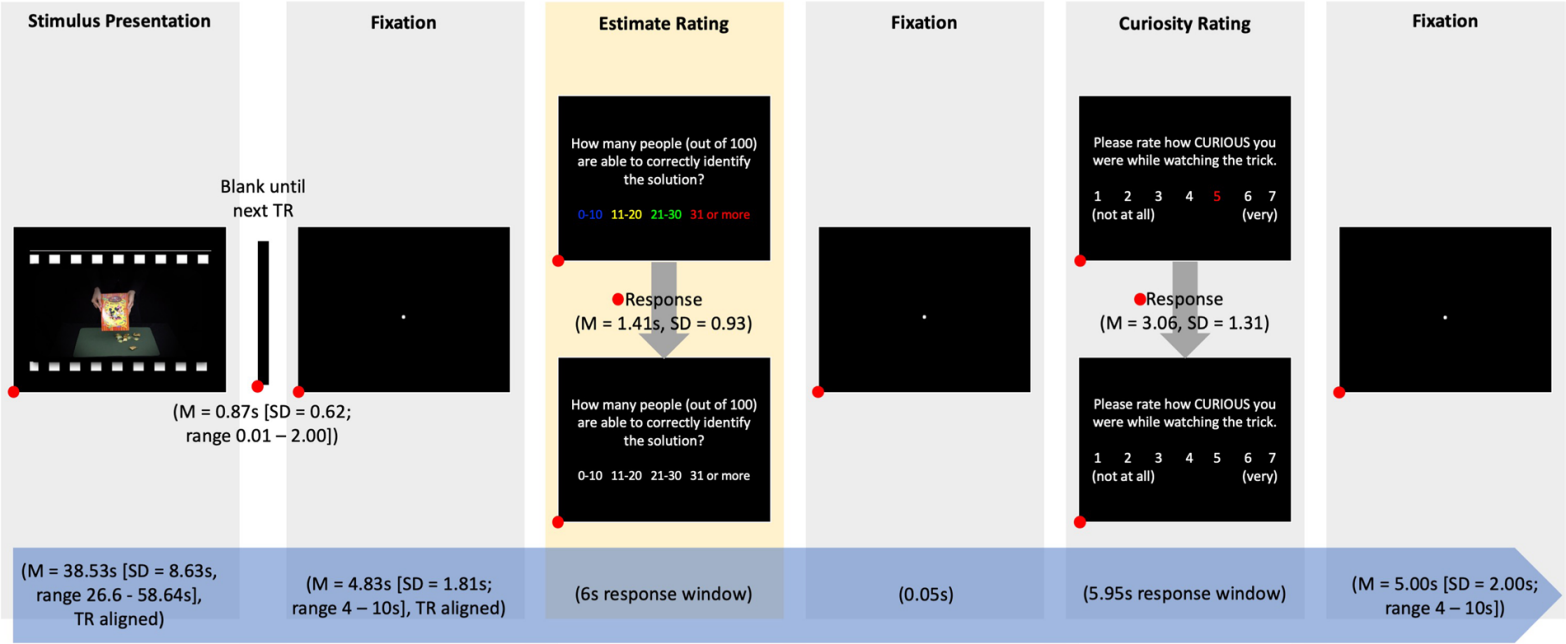
Magic Trick Watching Task. In each trial, participants viewed a magic trick and were asked to estimate how many people (out of 100) are able to find the solution (‘estimate rating’) and to rate their curiosity while watching the magic trick (‘curiosity rating’). The film framing surrounding the stimuli is for illustration purposes only and was not used in the experiment. The start of the magic trick presentation and the beginning of the fixation afterwards were aligned with the beginning of the acquisition of a volume (‘TR aligned’) by synchronisation with the scanner TTL pulse. A blank screen was presented between the end of the magic trick and the beginning of the next TR (i.e., start of fixation). Fixations were jittered using the same jitter intervals for all participants. All response windows were fixed. When a response was given before the end of the response window, the coloured font turned white, but the trial would not progress until the end of the response window. All time stamps collected are marked with red dots. While the trial structure looked identical for participants in both groups, participants in the experimental group were instructed that each correct response in the ‘estimate rating’ (highlighted in yellow) leads to an additional monetary bonus payment of £0.80.

During each trial (range of length 49.94–87.99 s, *M* = 63.29 s, *SD* = 8.71 s; see Fig. 2) in the incidental learning phase, participants were presented with a magic trick video and afterwards had to give an estimate as to (a) how many people (out of 100) would be able to correctly figure out the solution to this magic trick with answer options of ‘0–10’, ‘11–20’, ‘21–30’, and ‘31 and more’ (*‘estimate rating’* containing the incentives manipulation, highlighted in yellow, see below) and (b) how curious they were while watching the trick using a 7-point scale (1 = ‘not at all’, 7 = ‘very’; *‘curiosity rating’*). Note that participants’ subjective solutions were not recorded and likewise, objective solutions from the MagicCAT stimulus base were not presented to participants. We decided to use a 7-point scale for the curiosity ratings based on a previous fMRI study^18^ presenting magic trick videos from the MagicCAT stimulus set. Previous research showed that the number of response categories does not influence the proportion of format used (if more than 3 response categories are offered). Likewise, while 3- and 5-point scales are prone to increased use of the midpoint, the tendency of using the midpoint category decreases as the number of response options increases^37^. Therefore, we consider that using the 7-point scale would not introduce bias. In the MMC Dataset, participants used the midpoint (a rating of 4) response in 351 out of 1800 (or 19.5%) which was similar to their tendencies to use a rating of 3 (in 266 out of 1800 trials (or 14.8%)) or 5 (in 384 out of 1800 trials (or 21.3%)).

Responses were recorded using a four-button MRI-compatible response device (https://www.curdes.com/mainforp/responsedevices/buttonboxes/hhsc-1×4-cr.html) where the index finger would be on the first (i.e., blue) button, the middle finger on the second (i.e., yellow) button, the ring finger on the third (i.e., green) button and the small finger on the fourth (i.e., red) button. This was practised on a colour-coded keyboard outside the MRI scanner. The beginning of each magic trick presentation was in alignment with the beginning of the acquisition of a volume by synchronisation with the scanner TTL (transistor-transistor logic) pulse. Likewise, the fixation (white dot on black screen) presented for 4–10 s (jittered; *M* = 4.83 s, *SD* = 1.81 s) after the magic trick was aligned to sync with the TTL pulse and a blank screen was presented between the end of the magic trick and the fixation (*M* = 0.87 s, *SD* = 0.62 s, min = 0.01 s, max = 2.00 s). All timings (i.e., durations of jittered fixations and response windows) were set to be multiple of the repetition time (TR) of 2 s, thereby allowing the concatenation of the task time series in such a way that the order of magic trick videos remains invariant across participants^38^ despite pseudo-randomisation of the trials (see below).

For the estimate rating, each of the four answer options was presented in the colour corresponding to the finger on the button box that needed to be pressed to select a given option (i.e., ‘0–10’ in blue, ‘11–20’ in yellow, ‘21–30’ in green and ‘31 and more’ in red). The participants were given a fixed response window of 6 s to select an option. This response window was determined based on behavioural pilots^30,35^ where the median response time for the estimate rating was 4.41 s which was rounded to the next higher multiple of the TR. If they had made their choice before the end of the response window, the answer options changed from coloured to white and the screen was presented until the end of the response window followed by a fixation presented for 0.05 s. In 7 out of 1800 trials (or 0.39%), no response was given within the fixed response window of 6 s and the response was recorded as ‘NaN’ in the dataset.

Afterwards, the curiosity rating was presented with one number randomly highlighted in red. Participants were then asked to move the number to the left (pressing the blue button with the index finger) and/or to the right (pressing the yellow button with the middle finger) until the highlighted number corresponded to their curiosity which was confirmed by pressing the red button with the little finger. The response window was 5.95 s, again determined based on behavioural pilots^35^ where the median response for the curiosity rating was 3.38 s but accounting for longer reaction times based on how curiosity was assessed inside the scanner compared to the behavioural pilot studies conducted online. If an answer had been confirmed by pressing the red button before the end of the response window, all writing changed to white until the end of the trial. If the red button was not pressed before the end of the response window, the last number highlighted in red was recorded as curiosity rating together with a time stamp as the reaction time. Comparing the recorded response time of the curiosity rating with the response time window revealed that participants did not press the red button before the end of the response window in 119 out of 1800 trials (or 6.61%). The curiosity rating was again followed by a fixation presented for 4–10 s (jittered; *M* = 5.00 s, *SD* = 2.00 s) before the next trial started.

During each trial, we collected the estimate and curiosity responses as measurements as well as their respective reaction times. Additionally, we recorded the number that was randomly highlighted at the start of the curiosity rating as well as the number of clicks.

In total, 36 magic tricks were presented over three experimental blocks (i.e., 12 trials per block). The first trial in each block started with a fixation (2 s). To control for any effects of trial order and to ensure that any similarity in brain responses between participants can be attributed to similarity in curiosity ratings rather than to similarity in trial orders, the trial orders were pseudo-randomised. To achieve this, curiosity ratings from Ozono and colleagues^6^ were used to categorise each selected magic trick based on the median split as high vs low curiosity. In total, 25 trial orders (due to *N* per group = 25) were simulated in R^39^ so that high and low curiosity magic tricks were equally distributed across blocks (i.e., six each per block) while restricting the maximum number of consecutive tricks of either category to four. Additionally, the simulations were restricted so that the maximum range of Spearman-rank correlations of all pairwise trial orders did not exceed a threshold of .70. Each of the simulated trial orders was used once in the control and experimental group.

When presenting magic tricks inside the MRI scanner, Lau and colleagues^18^ analysed their data using a standard GLM approach defining the onset and duration of each magic trick. Likewise, specific features in each magic trick could be annotated and used as regressors in a GLM^26^. When trying to optimise the design for GLM-based analysis, it was not possible to do so for possible contrasts future researchers might be interested in based on what was presented in the magic trick (e.g., the occurrence of a third person supporting the magic trick by picking a card) or based on the curiosity rating and/or encoding performance. However, to support GLM-based analysis as much as possible, the design efficiency was maximised using AFNI’s(Cox, 1996) *‘@stim_analyze’* program for the contrast between the *video* (i.e., mock video and magic trick combined) and *ratings* (estimate and curiosity rating combined including fixation between both) by estimating the optimal jitter for the fixation following the magic trick video and curiosity rating, respectively. Firstly, 1000 random stimulus timings were produced (‘*make_random_timing.py’*). For that, we defined two ordered stimulus categories (video and rating) and their length (average magic trick file length = 38 s, length rating = 12 s based on the median response times observed in behavioural pilots^30,35^) as well as number of trials and blocks (i.e., twelve and three, respectively), block length (720 s to control for overall length of the MRI session based on budgetary constraints) and minimum (4 s based on specifications in previous fMRI studies presenting magic tricks^18^) and maximum (10 s to increase absolute uncertainty) rest. In a second step, the program evaluates the produced timings (‘*3dDeconvolve -nodata’*) to determine the iteration best suited to deconvolve overlapping hemodynamic responses with the smallest amount of unexplained variance (https://afni.nimh.nih.gov/pub/dist/HOWTO/howto/ht03_stim/html/AFNI_howto.html). The corresponding stimulus timings were used to calculate both jitter durations (i.e., fixation after watching the magic trick and fixation after the ratings) and the same values were used for all participants.

#### Memory task

**Fig. 3:**
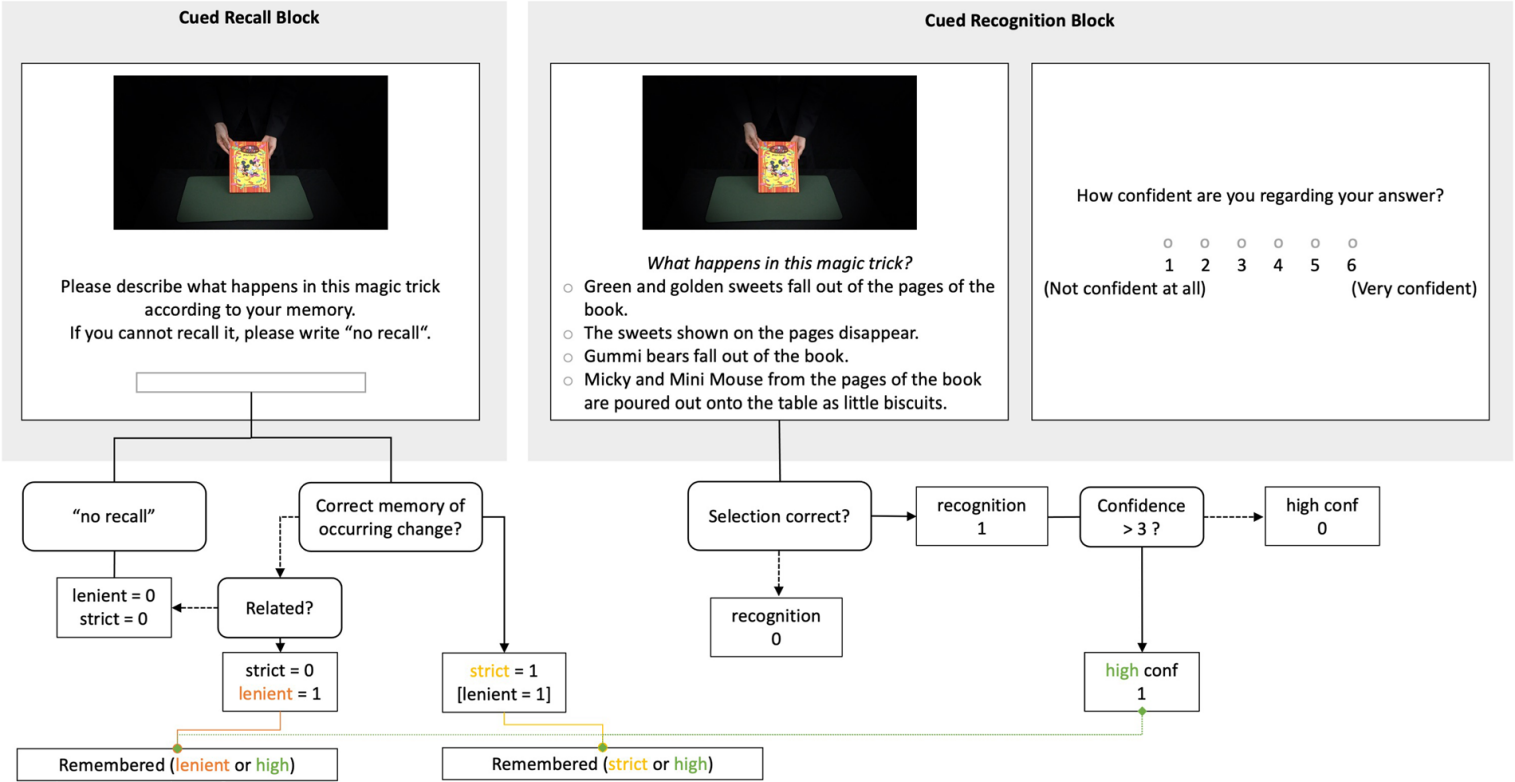
Memory Task and Coding of Answers. Memory for the magic tricks was tested using a cued recall (left side) and four-alternative forced-choice recognition (right side) paradigm, in a blocked manner (highlighted in grey). In the recall block, participants were presented with cue images and asked to describe what happened in the magic trick according to their memory. The recognition block used the same cue images and participants were asked to select one out of four options before rating their confidence. The lower part of the figure shows how both memory tasks were coded. Rectangles with rounded corners illustrate decision points and the inclusion of question marks highlights yes/no choices. The consequence of a ‘no answer’ is linked with dashed black arrows whereas consequences of ‘yes answers’ are connected by solid black arrows. Final decision outcomes are illustrated as rectangles with straight corners. In short, free answers from the cued recall paradigm were coded by the experimenters if participants did not write ‘no recall’. A magic trick was coded as recalled if the change that occurred was remembered. The selected answer from the recognition memory test was processed in a scripted manner by comparing the selected item against the corrected item. If an item was recognised, additional coding was performed based on two confidence thresholds (simultaneously). In a last step, the remembered criteria was coded based on whether the magic trick was either recalled (orange and yellow font/lines) or (indicated as vertical slash) recognised using recollection-based memory (dotted green lines and font).

Memory for all 36 magic tricks was tested using (1) a cued recall and (2) a four-alternative forced-choice recognition paradigm (see Fig. 3 upper half). During each cued recall trial, participants were presented with the cue image of a magic trick and asked to describe what has happened in this magic trick according to their memory using a free answer format text input. Participants were informed that their answers would be used to categorise whether they recalled a given magic trick and asked them to be as specific and descriptive as possible. Additionally, they were instructed to write ‘no recall’ if they could not recall it.

During the recognition task, each trial started with the presentation of the same cue image paired with the question ‘What happens in this magic trick’ and four verbal answer options (see Supplementary Information) presented in random order. Participants were asked to select one of the options and to afterwards rate their confidence on a scale from 1 (‘not confident at all’) to 6 (‘very confident’). For both answers, their response times were collected.

Participants’ recall for all magic tricks was tested before testing their recognition. Both assessments were self-paced and the cue images for each magic trick were displayed in independent, random order.

#### Working memory tasks

To measure working memory, we used the Corsi block tapping task^40,41^ and a modified version of the 2-back task^42^, both available in the PsyToolkit^43,44^ library (https://www.psytoolkit.org/experiment-library/). The Corsi Block Tapping task is a measurement of immediate spatial memory^40^. The participants saw nine cubes (impartially arranged) that were highlighted in sequence starting with two cubes. Immediately afterwards, the participants had to click on the cubes in exactly the same order. When successful, the next trial had the next higher number of cubes, otherwise, there was one more attempt for another sequence with the same number of cubes. The Corsi span was determined as the maximum number of cubes the participant was able to click on in correct order.

The 2-back task was also used to measure working memory^45,46^ (but see also^47^). Participants were shown a sequence of letters on the screen (each for max 2000 ms; 15 different letters used) and were asked to decide whether they saw the same letter two trials ago (i.e., *n* = 2 back). If the participants thought they saw the same letter, they had to press ‘M’ (for ‘memory’), otherwise, they were asked to press ‘N’ (for ‘no’). The task started with a practice block in which feedback was provided followed by four task blocks without feedback. Each block consisted of 20 trials.

#### Questionnaires

Throughout the data collection, different questionnaires were given to the participants. The whole questionnaire battery can be found in the supplementary information.

Constructs relevant in the context of curiosity, incentives and incentives-motivated learning were accessed in the pre-scanning session. Questionnaires included the Behavioural Inhibition and Behavioural Activation Scales (BIS/BAS)^48^ (20 items on a 4-point Likert scale ranging from 1 = ‘very false for me’ to 4 = ‘very true for me’), Need for Cognition^49^ (18 items on a 9-point Likert scale ranging from −4 = ‘very strong disagreement’ to + 4 = ‘very strong agreement’), Fear of Failure^50^ (9 items on a 5-point Likert scale ranging from 1 = ‘strongly disagree’ to 5 = ‘strongly agree’), Approach and Avoidance Temperament^51^ (12 items on a 7-point Likert scale ranging from 1 = ‘strongly disagree’ to 7 = ‘strongly agree’), and Trait Curiosity^52^ (18 items on a 4-point Likert scale ranging from 1 = ‘almost never’ to 4 = ‘almost always’).

Additionally, at the end of the scanning session, a task motivation inventory (intrinsic motivation^53^, task engagement^53^, interest^54^, boredom^55^, effort^56^, and pressure^56^) as well as task compliance and whether they were able to see the magic tricks properly were assessed inside the MRI scanner while we acquired the anatomical scan. The item order was randomised, and participants could answer on a 7-point Likert scale from 1 (‘definitely disagree’) to 7 (‘definitely agree’). Similar to the curiosity ratings, a random number was highlighted in red, and participants were asked to move the number to the left or right until the highlighted number corresponded to their rating. Outside the scanner, State Curiosity^52^ (20 items on a 4-point Likert scale ranging from 1 = ‘not at all’ to 4 = ‘very much so’) was measured together with sleep and alcohol consumption in the last 24 hours. Moreover, we asked the experimental group about their reward expectations and whether they invested effort to increase their rewards (7-point Likert scale from 1 = ‘strongly disagree’ to 7 = ‘strongly agree’) and how much reward they expected to have earned (free text answer). As a manipulation check, we asked all participants to describe the hypothesis of the experiment.

At the end of the memory tests, a final questionnaire was administered to determine whether participants were aware of their memory being tested and whether they intended to encode the magic trick while watching them on a 6-point Likert scale (6 = ‘definitely agree’, 1 = ‘definitely disagree’). Additionally, participants in the experimental group were asked whether they believed the incentives manipulation. Lastly, we asked about any internet connection problems as well as whether participants had any experience producing magic tricks on a 6-point Likert scale (1 = ‘never’, 6 = ‘very frequently’).

### Experimental procedure

To be able to investigate incidental encoding (similar to previous research^32,57^), participants were presented with a cover story that the study investigates problem solving, social cognition, and the associated brain processes. They were briefed that they would be performing a viewing and judgement task involving magic tricks inside the MRI scanner and that there would be an online follow-up assessment related to their responses a week later. They were scheduled for all sessions of data collection: (1) a pre-scanning online session, (2) MRI session in the lab and (3) an online surprise memory test a week after scanning (see Fig. 1a).

#### Pre-scanning session

Participants were instructed to follow a weblink (https://www.psytoolkit.org/cgi-bin/psy2.5.3/survey?s=Y9aCu) in order to take part in the session. The link directed them to an online assessment implemented using PsyToolkit^43,44^ version 2.5.3 assessing demographics, current and lifetime disease diagnoses, MRI safety criteria, questionnaires, and working memory measurements. There were on average 6 days (*SD* = 11d 7 h 55 min, *median* = 1d 20 h 9 min, *min *= 2 min, *max* = 66d 18 h 17 min) between the online assessment and the MRI session. Participants’ median completion time was 25.5 min (*M* = 89.3, *SD* = 274.74, *median* = 25.5, *min* = 16, *max* = 1532). High values in mean and SD compared to median suggest that some participants could have left the screen open after the assessments or took breaks in between. By looking at the completion time in more detail, it took participants on average 4.8 min (*SD* = 6.13, *median* = 3.64, *min* = 1.22, *max* = 44.41) to provide personal information and on average 34.89 min (*SD* = 168.6, *median* = 8.89, *min* = 1.55, *max* = 1202.2) to complete the questionnaires. No information is available regarding how long participants took to complete the working memory tasks themselves, however. The information introducing to the Corsi task was displayed for on average 2.79 min (*SD* = 10.68, *median* = 0.54, *min* = 0.02, *max* = 58.84) and information introducing the 2-back task was viewed for on average 0.73 min (*SD* = 0.5, *median* = 0.59, *min* = 0.02, *max* = 2.33).

#### MRI session

The MRI experiment was divided into the following critical stages: (a) participant preparation and practice, (b) a pre-learning rest phase, (c) an incentives and/or curiosity motivated incidental learning phase, (d) a post-learning rest phase, and (e) a post-experimental assessment of intrinsic motivation and state curiosity (see Fig. 1a, lower half outlined in dotted line). The experiment has been presented using PsychophysicsToolbox (PTB) 3^58^ with GStreamer media framework run on Matlab (R2018b) on a 13 inch Apple MacBook Pro (2018) that was connected to the 32inch back-projection screen (BOLDScreen, Cambridge Research Systems LTD., UK) mounted at the head end of the scanner bore in the MRI suite. Participants looked at the screen through an eye-tracking sensitive mirror attached to the head coil. The MRI session lasted approximately 2 hours. An exact record of the instructions that were presented to the participants can be found in the supplementary information.

##### Participant preparation and practice

Upon arrival in the MRI area, the participants were demetalled and underwent two practice trials of the magic trick watching task. The practice trials were presented using PsychophysicsToolbox 3^58^ with GStreamer media framework run on Matlab (R2016a) on a 13 inch MacBook Air (2013). The responses were collected using the laptop keyboard that had the letters H, J, K, and L marked in blue, yellow, red, and green, respectively. Participants were given the opportunity to ask questions before they were accompanied to the MRI suite by MRI-trained experimenters (SM & CPM). There, they were introduced to the button box before inserting noise-cancelling earplugs. Next, participants were placed on the scanner bed and pillows were placed under their knees and on either side of their heads for comfort and to minimise movements during the scan. Participants held the button box in their right hand and were given a bulb into their left hand that they could squeeze in case of an emergency. They were instructed to not cross their arms or legs at any time and to try to keep as still as possible through the duration of the scan, but that they could ‘wiggle their arms and legs’ in between scans when the experimenters communicated with them through the intercom. After being moved to the isocentre, localizer and field map were acquired whilst the participant read through the instructions for the resting phase.

##### Pre- and post-learning rest phases

Similar to previous studies^59^, we implemented pre- and post-learning rest phases to measure post-encoding and consolidation processes. During the rest phases (10 min each), participants were laying in the scanner and were instructed to keep as still as possible with eyes open while presented with a white screen (without fixation). They were further asked to blink as usual and to try to not think about anything. These instructions were presented in written form on the screen and also repeated by the experimenter through the intercom.

##### Incentives- and/or curiosity-motivated incidental learning phase

After the pre-learning rest phase, participants were asked to read again through the instructions of the magic trick watching task whilst a field map was acquired. For the experimental group only, the instructions included a statement that participants could earn an additional bonus payment of up to 50% of their compensation: each correct answer to the question of how many people would be able to find the solution (estimation rating in Fig. 2, highlighted in yellow) translated into a bonus payment of £0.80 (50% additional bonus payment should have translated to £0.40 per correct answer). We made a calculation error here. However, no participant reported to notice this error. This information was highlighted in green and had to be confirmed by pressing the third (i.e., green) button on the button box. The control group, however, did not receive such information. In each of the three task blocks, 12 magic trick videos were presented. Due to variation in the length of the video clips and their randomisation, each block lasted on average 12.66 min (*SD* = 0.41 min, range in min = 11.67–13.77). In between the task blocks, participants were offered a break at the end of which participants in the experimental group were reminded of the possibility of additional bonus payments. Before the start of each block, the experimenters talked with the participants through the intercom to ensure that the participants knew what to expect and what to do. The incentives- and/or curiosity-motivated incidental learning phase lasted on average 37.98 min (*SD* = 0.02; range in min = 37.97–38) plus breaks.

##### Post-experimental assessments

After the post-learning rest phase, the anatomical scan was acquired. During the sequence, participants completed the task motivation inventory described above. The questionnaire was self-paced. If participants finished the questionnaire before the end of the anatomical sequence, they were asked to stay still until the end of the scan after which they were removed from the MRI scanner. Outside the MRI scanner, participants filled in another questionnaire as well as some follow-up questions, again implemented using PsyToolkit^43,44^ version 2.5.3 (https://www.psytoolkit.org/cgi-bin/psy2.5.3/survey?s=JDPGx).

#### Surprise memory session

Approximately one week after scanning (range = [6d 19 h 55 min; 9d 11 h 18 min], *M* = 7d 10 h 19 min, *SD* = 13 h 41 min), participants took part in a surprise memory test online (consisting of cued recall and recognition) implemented using a developmental version of Collector^60^. They were asked to do this experiment roughly around the same time as they participated in the MRI experiment in the lab and were reminded two days in advance via email. Upon completion of the memory task, participants filled in a short questionnaire (see Supplementary Information) about, e.g., whether they were aware that their memory would be tested. Afterwards, they were debriefed about the scope of the study and the between-group incentives manipulation. All participants received a bonus payment of £7.20. Participants took on average 47.98 min (*SD* = 42.81, *median* = 35.05, *min* = 17.09, *max* = 309.37) to complete the assessment. The recall block lasted for on average 20.68 min (*SD* = 13.66, *median* = 17.09, *min* = 4.93, *max* = 74.11) and the recall block lasted on average 14.14 min (*SD* = 5.65, *median* = 12.46, *min* = 6.42, *max* = 30.74). Due to software development, the original link cannot be assessed anymore. However, we re-created the experiment as close to the original version as possible here https://magic-memory-curiosity.github.io/fmri/App/Run.html?platform=github&location=magicmemory_fmri_redo&name=condition_1 and a record of instructions presented to the participants can be found in the supplementary information.

### Coding of memory measurements

Data collected in the cued recall block of the memory test was coded using dummy coding (1 = recalled, 0 = not recalled) in two steps: firstly, R^39^ was used to automatically assign a zero to all answers containing ‘no recall’ and variations thereof. In the second step, a trained rater (CPM) coded all answers manually. The coding was performed for each trick separately to apply the same standard across participants and reviewed for consistency on participant level. Any cases requiring further attention or correction were flagged up and resolved after discussion (with KM). For a trick to be coded as recalled, it was essential that the change that occurred was remembered, however, minor details could be wrong. Strict and lenient criteria were applied. The strict criterion captures whether the participants referred correctly to the change that occurred. If they recalled something related to the change that occurred without correctly specifying the change that occurred, a 1 would be assigned to the lenient, but not the strict criterion, hence reflecting gist-based memory. For instance, let us assume the magician changes the colour of the back of the card from blue to red (as done in the trick with stimulus id K21_long, see Supplementary Information for a more detailed description). If the participants wrote that the magician changed the colour of the cards, but used the wrong colours, they would still fulfil the strict criteria (and hence, lenient as well). However, if the participants remembered that the appearance of the cards was changed without recalling that it was the colour that changed, they would fulfil the lenient criteria as this is related to the actual change but would not pass on the strict criteria as the actual change was not recalled. In comparison, the trick would be classified as not recalled on both criteria if the participant had written that the cards disappeared. Overall, the coding of recall performance on the lenient compared to strict criteria only differed in 81 out of 1800 trials (or 4.5%).

To code the recognition performance, the response was compared to the correct answer and coded with 1 if they were the same and with zero if they differed. Additionally, we combined recognition and confidence to measure recollection-based recognition^61,62^ using ‘high confidence recognition’ (i.e., recognised with a raw confidence rating larger than 3).

Similar to previous studies investigating memory encoding using naturalistic stimuli^63^, we also combined recall and recollection-based recognition in which a trick is classified as remembered if participants succeeded in either of the cued recall (lenient vs. strict) or recollection-based recognition criteria, hence creating another two memory thresholds (remembered (lenient or high) and remembered (strict or high)). All coding procedures are illustrated in the lower half of Fig. 3.

For the 2-back task, responses in the four task blocks were used to determine the number of observations in each cell of the stimulus-response matrix according to Signal Detection Theory^64^. Additionally, hit rate (n _hits_/n _n-back trials_) and false alarm rate (n _false alarms_/n _non n-back trials_) were computed to derive accuracy (or discrimination index; hit rate - false alarm rate)^46,65^.

### MRI acquisition

We used a Siemens Magnetom Prisma_ft 3.0 T scanner (software syngo MR E11) with a 32-channel head matrix coil to acquire anatomical and functional images at the Centre for Integrative Neuroscience and Neurodynamics (CINN), University of Reading, during a single 90-min scanning session.

Functional whole brain images were acquired using a T2*-weighted gradient-echo echo-planar imaging (EPI) pulse GRAPPA single-band acquisition sequence (sequence name: _epfid2d1_64, sequence variant: SK, single-band acquisition) with 37 axial slices (in-plane resolution of 3 × 3 × 3 mm, interslice gap of 0.75 mm), interleaved in transversal direction, co-planar with anterior-posterior commissure line, ascending from bottom to top (echo time (TE): 30 ms; TR: 2000 ms; flip angle (FA): 90°; field of view (FOV): 192 × 192 mm^2^; in-plane matrix: 64 × 64; phase encoding direction: P >> A). These scanning parameters are the same as used by Lau and colleagues^18^ who also presented magic tricks from the MagicCAT stimulus set. The rest phases were acquired in one run each containing 300 volumes after which the scanner stopped automatically. During the task, 1140 volumes were acquired over three runs (run 1: *M* = 379.88, *SD* = 14.73; run 2: *M* = 377.84, *SD* = 10.57; run 3: *M* = 381.64, *SD* = 10.88) and the scanner was stopped manually at the end of each run. Slices were positioned to cover the whole brain based on the localiser scan and slice positioning from the first resting-state scan was used as reference and copied to all following acquisitions. For individuals with large heads, superior parts of the brain were prioritised so that the most inferior slices of the cerebellum are missing for some participants (see ‘Brain coverage’ below).

B_0_ data were acquired on the same image matrix and the same geometric prescription as the functional data, using a dual-TE 2D gradient-echo sequence (sequence name: _fm2d2r, sequence variant: SP) with the following parameters: TR = 488 ms, TE1/TE2 = 5.19/7.65 ms, FA = 60°.

A high-resolution T1-weighted three-dimensional anatomical image was collected using an MPRAGE-gradient sequence (sequence name: _tfl3d1_16ns, sequence variant: SK_SP_MP) with 192 × 1 mm slices (in-plane resolution of 1 × 1 × 1 mm; TE: 2.29 ms; TR: 2300 ms; inversion time (TI): 900 ms; FOV: 192 × 256 mm^2^; in-plane matrix: 192 × 256; FA: 8°).

During the scanning, participants were monitored using a camera and an eye tracker to ensure that participants attended all stimuli and kept their eyes open during rest phases. However, no eye-tracking data was collected. Further, scanning output was monitored throughout acquisition to ensure high quality of the data. The structural scans were manually reviewed for incidental findings.

### MRI preprocessing

MRI data were converted from dicom to Neuroimaging Informatics Technology Initiative (NIfTI) format and into Brain Imaging Data Structure (https://bids.neuroimaging.io/) using pyBIDSconv v1.1.9 (https://github.com/DrMichaelLindner/pyBIDSconv) and preprocessed using the AFNI (version 21.2.03) software suite^66^, unless indicated differently, with specific AFNI programs indicated using parentheses. The outputs of all preprocessing steps were inspected manually.

#### Anatomical

The anatomical scans were segmented, parcellated and inflated with FreeSurfer^67,68^ version 6.0.0 using *‘recon-all’* and default parameters. For three participants (ID 7, 35, and 37), white matter or pial surface were edited manually. FreeSurfer output was converted to NIfTI using AFNI’s (version 21.0.02) *@SUMA_Make_Spec_FS* to create tissue masks for the lateral ventricle (VENT) and white matter (WM; *‘adjunct_suma_fs_mask_and_qc’*). The grey matter (GM) segmentations based on the Desikan-Kiliany cortical atlas^69^ were used to create the GM mask (‘*3dcalc’*). Cardinalised FreeSurfer output was obliquified to match the affine matrix of the raw anatomical images.

In a separate step, the raw anatomical images were uniformized, anisotropically smoothed, ceiling-clipped, skull-stripped in two iterations and non-linearly registered to ICBM 2009c Nonlinear Asymmetric Template^70^ using *@SSWarper*. The affine matrix applied to transform the original dataset to the template was saved to use for the functional preprocessing (see below).

#### Functional

##### Post-acquisition processing

During the magic trick watching task, the scanner was stopped manually at the end of each task block because the pseudo-randomised presentation of the magic trick video stimuli caused the length of each block to vary across participants. During resting-state acquisition, however, the scanner was stopped automatically after 10 min (300 volumes). To discard any volumes that were acquired after the task had stopped, but before the scanner was stopped, we cut (using *‘3dTcat’*) each functional task run to the total block length (i.e., the end of the fixation after the curiosity rating for the twelfth magic trick presented in this block). This was important not only because the volumes acquired after the task block had ended could have been subject to increased head motion and hence, biased data quality assessments. Additionally, this ensures that the data has the same length for all participants for later validation analysis (see below). The cut timeseries are regarded as raw data and shared on OpenNeuro.

##### Minimal preprocessing pipeline

For part of the data quality assessment, minimal preprocessing was applied to each EPI timeseries separately to avoid additional interpolation associated with cross-run alignments using *afni_proc.py*. The pipeline consisted of despiking, slice-timing and head-motion correction, and intrasubject alignment between skull-stripped anatomical and functional scans (see Fig. 1b). The latter two steps both used a low-motion volume (i.e., the volume with the lowest outlier fraction determined based on respective EPI timeseries data) and transformations were combined into one interpolation. This pipeline was used to determine brain coverage, head motion, and temporal signal-to-noise ratio (tSNR, option *‘-volreg_compute_tsnr’*).

##### Full preprocessing pipeline

In addition to the minimal preprocessing, full preprocessing was applied to the data in a separate pipeline. As a first step, the EPI timeseries were distortion-corrected along the encoding axis (P >> A) using the phase difference map (*‘epi_b0_correct.py’*)^71^. The resulting distortion-corrected EPIs were then processed separately for each task, but scans from the same task were processed together. The same blocks were applied to both task and resting-state distortion-corrected EPI data using *afni_proc.py*: despiking, slice-timing and head-motion correction, intrasubject alignment between anatomy and EPI, intersubject registration to MNI, masking, smoothing, scaling, and denoising (see Fig. 1b).

Head-motion correction to a low-motion volume (again, based on outlier fraction) was carried out within each run, adding a cross-run registration step to align each within-run base to the volume used for intra-subject alignment (*‘-volreg_post_vr_allin yes -volreg_pvra_base_index MIN_OUTLIER’*). Intrasubject alignment was based on the skull-stripped anatomical image created using *@SSWarper* and the low-motion volume from the first run. Intersubject registration to the ICBM 2009c Nonlinear Asymmetric Template was achieved using non-linear transformation applying the affine matrix computed using *@SSWarper*. Due to variation in slice coverage (see ‘Brain coverage’ below), the extent mask (a mask of voxels that have valid data for every TR) was not applied (*‘-volreg_no_extent_mask’*). Transformations for motion correction and intra- and intersubject alignment were concatenated and applied in a single step to avoid repeated resampling and interpolation. To eliminate additional spatial effects induced by non-linear transformations potentially resulting in diversity of spatial correlations across voxels^72^, data was spatially smoothed to *achieve* a fixed, global smoothness with full-width half maximum kernel (FWHM) of 8 mm (using *‘-blur_to_fwhm -blur_size 8’*). The chosen kernel mirrors recommendations for the smoothness of data in intersubject correlation (ISC) to be slightly more than twice the voxel size^73^ and was also applied to the resting state data in the interest of consistency. Finally, time series were scaled to a mean of 100.

The tissue maps created using FreeSurfer were resampled to EPI resolution and eroded before applying them to create local WM regressors using fast ANATICOR^74^ and to derive the first three principal components (PC) of the VENT. Those together with two Legendre polynomials, six demeaned (per run) motion parameters and the derivatives of the motion parameters were included as regressors with VENT PC and motion regressors for each run used as separate regressors, hence allowing the magnitude of the regressors to vary over time. Bandpass filtering was performed during regression (*‘-regress_bandpass 0.01 1’*). The combination of two Legendre polynomials and bandpass filtering was recommended as the most suitable option (please see https://afni.nimh.nih.gov/afni/community/board/read.php?1,165243,165256#msg-165256 for a detailed discussion). Time points were censored if motion (Euclidean norm of motion derivatives) exceeded 0.3 or if ≥ 10% of the brain were outliers^75^. Censored timepoints were not removed but set to zero to preserve the temporal structure of the data. This pipeline implements the latest guidance for preprocessing of resting state data^76^ and follows previous work^75,77^ applying similar analysis methods as used in this publication.

Preprocessing was validated by manual inspection of the single participant html quality report that was created by *afni_proc.py* visualising volumes, intra- and intersubject registration as well as seed-based correlation maps. To identify any outliers after preprocessing, ‘*gen_ss_review_table.py’* was used to generate a table with the basic summary quantities from preprocessing. Additionally, we implemented checks for left-right orientation errors^78^ (using *‘-align_opts_aea -check_flip’*). The final output of preprocessing were denoised timeseries with 1140 and 600 volumes, respectively, which were used for the basic fMRI validation analysis (ISC and seed-based functional connectivity analysis).

### Basic data validation analyses

All behavioural analyses were carried out using R^39^ version 3.6.3.

#### Timing

Due to the naturalistic nature of the stimuli used in this study, standard GLM analysis approaches based on modelling the BOLD response with respect to onset and duration of the stimuli may not be fully suited and model-free approaches like ISC often used in the context of naturalistic fMRI can be preferable. To allow for the rationale of correlating voxel-wise time series across participants, the visual input must be held constant across participants and time-locked with the TR. This is why the beginnings of crucial events (i.e., video stimulus presentation and fixation afterwards) were time-locked with the TR and why jitter durations and response windows were equal to (or multiples of) the TR. To be able to validate stimulus timing, observed durations (computed using timestamps collected during experiment presentation) were compared to their intended (i.e., programmed) duration.

#### Variance decomposition

To address the question whether the MMC Dataset is suitable to investigate within-person variability in curiosity, confidence, and memory encoding, mixed-effects models were applied to decompose the data into three different variance components^6,79^: *participant variance* captures overall individual differences between participants (i.e., a participant might have a high overall memory performance or generally gives high curiosity ratings for all magic tricks), whereas *stimulus variance* reflects differences between the magic tricks (i.e., a trick was very easily encoded by all participants or generally rated low on curiosity). Additionally, *participant x stimulus variance* represents individual differences in participants’ responses to different stimuli hence indicating that a participant encoded/was curious about a magic trick due to one’s specific conditions and preferences. While participant x stimulus variance serves as a proxy for within-person variability, it also includes variance from measurement errors that cannot be separated statistically.

In the context of binomial data like memory encoding, a simple variance decomposition is not available. However, if the underlying probabilities are not extreme, the binomial data can be treated as if it was normally distributed to get an approximate indication for the variance decomposition^80^. Given the non-extreme nature of the encoding probabilities in the MMC Dataset, the same mixed effects model (dependent variable ~ (1|participant id) + (1|stimulus id)) was applied to all dependent variables using the *lme4* package^81^ version 1.1.26.

#### MRI data quality assessment using MRIQC

In addition to the preliminary quality control (QC) during the scanning, several other data quality assessments were carried out. Raw data of all participants were inspected manually. However, because manual QC is prone to subjectivity and human error, we additionally used MRIQC^82,83^ to derive a variety of image quality metrics (IQMs) based on the raw NIfTI scans in a standardised manner.

MRIQC^82,83^ (version 0.16.1) was deployed using Docker (version 20.10.16). MRIQC implements a nipype workflow to apply minimal preprocessing to individual anatomical and functional scans before extracting IQMs (one value per IQM per scan). IQMs for anatomical data can be grouped into four broad categories^82,84^: measures based on noise measures, measures based on information theory, measures targeting specific artefacts, and other measures. IQMs for functional data^84^ include some of the same spatial measures used for the anatomical scans as well as measures of temporal information (e.g., tSNR), and measures targeting specific artefacts like head motion (e.g., framewise displacement^85^) as well as IQMs from AFNI.

In addition to a summary JSON file (per participant for anatomical data or per scan for functional data) containing the IQMs, a comprehensive html file is created including reports for visual assessments that were reviewed manually. Additionally, group reports are created separately for anatomical and functional modality. These include a table with all IQMs for each scan as well as an interactive group html report that was reviewed for outliers.

Due to the fact that many IQMs are no-reference metrics^86^, context is provided for both, anatomical and functional data by plotting IQMs observed in our dataset against those of crowdsourced MRIQC IQMs from the MRIQC Web-API^84^ using a custom-made R markdown script.

In the MMC Dataset, there are in total five functional scans, two stemming from resting-state and three from the magic trick watching task. IQMs for functional scan were extracted for further examination for task- and group related patterns using a custom-made R markdown script to create different plots and summary statistics of IQMs averaged within participants after standardising each IQM. Additionally, linear-mixed effects (LME) models (IQM ~ group + task + (1|ID) + (1|acquisition) with acquisition = three magic trick watching plus two resting-state scans) were applied to the IQMs to test for group and task (i.e., difference in IQM in the magic trick watching task compared to resting-state). Bonferroni correction (α = 0.05/*n*_IQMs_) was applied. In total, there were 37 IQMs derived from the functional scans, leading to a Bonferroni-corrected p-value of α = 0.05/37 = 0.0014 for the LME analyses. If data quality is not affected by task or group, we would expect to not find significant main effects here.

#### Brain coverage

To quantify the brain coverage in each functional scan, a brain mask was created based on FreeSurfer parcellation excluding the brain stem and slightly dilated to fill holes (*‘3dcalc’* and *‘3dmask_tool’*). This mask was intersected with the EPI mask created by afni_proc.py (*‘3dAutomask’*). A box was fitted for each of the resulting two masks to determine its extent in slices (‘3dAutobox’) and their difference in slices in inferior-superior direction was calculated (see Aliko *et al*.^87^ for a similar approach to determine cerebellar coverage).

#### Head motion

Head motion is a common artefact in fMRI data^85^. While MRIQC quantifies head motion as framewise displacement (FD), the information extractable is limited to the mean value for each scan as well as number and percentage of volumes above a given threshold (specified as 0.5 mm). To be able to further investigate and plot FD distributions, motion estimates for each scan created during minimal preprocessing were used to calculate FD following the definition by Power *et al*.^85^: after transforming degree measurements for roll, pitch, and yaw into mm assuming a 50 mm head radius, motion derivatives were calculated and the six parameters were summarised into one scalar per volume. As a next step, mean FD was calculated for each scan and then averaged for each participant to create a per-participant summary metric to quantify the amount of head motion in the sample.

#### Temporal signal-to-noise ratio (tSNR)

To assess image quality, tSNR is often used as it captures the ratio of mean signal to the standard deviation of noise in a timeseries^88^. In a similar manner to FD, tSNR is calculated automatically by MRIQC, but only the median value for each scan is included as output. To inspect tSNR values in a similar way as done for FD, data from the minimal preprocessing pipeline has been used. More specifically, by specifying *‘-volreg_compute_tsnr’* tSNR maps were created automatically using the despiked, slice timing and head motion corrected EPI timeseries that were aligned to the anatomical scan to compute the mean and dividing it by the standard deviation (the latter after detrending). To create a GM mask to extract the tSNR values for each voxel, the FreeSurfer whole brain parcellation was resampled to EPI resolution, binarised, and dilated to fill any holes (*‘3dmask_tool’*), then ventricle and white matter were removed (*‘3dcalc’*). The extracted values were averaged within each participant separately for each scan before computing the mean within each participant.

To further quantify tSNR across the whole brain, the tSNR maps automatically created during the full preprocessing pipeline were used. The timeseries from all runs for each task were combined to calculate the mean of the total timeseries before the nuisance regression step. The mean was divided by the standard deviation of the timeseries after nuisance regression. These maps were then used as input for a One-Sample *t*-test (carried out separately for task and rest with ‘*3dttest*++ *-Clustsim*’) and the resulting map was Bonferroni-corrected for multiple comparisons (see section on ‘Thresholding’ for details). Comparing the resulting maps (‘*3ddot*’) showed high similarity (*r*_mean tSNR_ = .997; *r*_z values_ = .999), so that maps were averaged within each participant before re-computing the One Sample *t*-test. To determine whether there were any group effects, the within-participant averaged tSNR maps were also included in a Two-Sample *t*-test separately applying the same thresholding as described above.

#### Intersubject correlation (ISC)

When presenting naturalistic stimuli to participants, data quality can be demonstrated using ISC analysis to estimate the extent of synchronised neural response to the stimuli^87,89,90^. In ISC analysis, a data-driven and model-free approach, the time course of each voxel is correlated for each pair of participants to create pairwise ISC maps where the voxel value reflects the correlation^27^. By computing correlations across pairs of participants, the stimulus-driven (and hence time-locked) extrinsic component of the BOLD response determines the level of synchronicity in the voxel-wise time courses across participants while the internally-fluctuating intrinsic component is usually cancelled out as noise^28^. During the magic trick watching task, participants were asked to view and rate the presented magic trick stimuli. Because neural responses during ratings are more likely to be driven by higher cognitive functions involved in the decision-making process rather than by what was presented on the screen and because response times varied between participants, ISC analysis focused on volumes acquired during magic trick presentation.

The fully preprocessed and denoised BOLD task timeseries (1140 volumes) were concatenated (again using *‘3dTcat’*). This step was necessary to remove all volumes acquired during the fixations, mock video presentation, and estimate and curiosity rating as well as to re-order the volumes acquired during pseudo-randomised stimulus presentation so that all volumes acquired during magic trick presentation are in the same order. Such steps have previously been performed by others^38^ presenting short video clips in pseudo-randomised order before calculating ISC maps. Onset and duration were converted from seconds into volumes using the formula volumes = ceil(round(time in secs)/TR) to select volumes acquired during the magic trick presentation. Due to non-systematic variation in the duration of the magic trick presentation (stimulus-wise computed deviation in seconds ranging from 0.00–0.14 with *M* = 0.06 and *SD* = 0.05; see ‘Timing’ below), the average display duration of each magic trick was used to determine the duration to ensure that the concatenated timeseries will have the same number of volumes for all participants. The actual length of magic trick presentation differed slightly from the average length (range of absolute difference in seconds: 0–0.6, *M* = 0.02, *SD* = 0.07); however, after converting, the average duration in volume differed from the actual duration in volume in only 49 out of 1800 (or 2.72%) total trials.

The final concatenated task timeseries (594 volumes) was used to compute pairwise Pearson correlations between each pair of participants for each voxel (using *‘3dTcorrelate’*). This resulted in ($\frac{1}{2}N\ast (N-1)=1225$ pairwise correlation maps that were Fisher’s z transformed. To determine brain areas that exhibited a significant ISC, a voxel-wise linear mixed effects model with crossed random effects accounting for the interrelatedness of the pairwise ISC maps^77^ was applied (*‘3dISC’*).

#### Seed-based resting-state functional connectivity (sFC)

In seed-based functional connectivity (sFC) analysis, the time course of a seed region - an *a priori* region-of-interest - is extracted and correlated with the time course of all other voxels within the brain to show the regions or networks that are most strongly functionally correlated with the seed^91–93^. This model-based approach sheds light on the question to which network a specific seed or region belongs to. Based on resting-state data from 1000 participants, Yeo and colleagues^94^ proposed a coarse parcellation of the human cortex into seven networks (e.g., visual network, default mode network). The knowledge of these resting-state networks allows us to use sFC as a validation analysis for the fMRI acquired during rest: if a seed region within a given network is used to perform sFC, the resulting map of regions exhibiting a high correlation should show overlap with the network the seed region is part of, thereby replicating previously established networks. In fact, AFNI uses this approach as part of their automated quality control within *afni_proc.py*: in the absence of any stimuli files, sFC is automatically calculated for each participant using three seeds and a 6 mm sphere around them in the left precuneus (MNI coordinates 5 L, 49 P, 40 S), the right primary visual cortex (MNI coordinates 4 R, 91 P, 3 I), and the left auditory association cortex (MNI coordinates 64 L, 12 P, 2 S) and plotted for each participant separately thresholded at *r* ≤ −.3 and *r* ≥ .3.

To replicate these analyses for the whole sample, the fully preprocessed and denoised resting-state timeseries concatenated across both runs were prepared for sFC analysis using *‘3dSetupGroupInCorr’* whilst restricting the volumes with an average slightly dilated GM masks averaged across the sample. The resulting data file containing all timeseries within the mask from all datasets was passed to *‘3dGroupInCorr’* together with the three seeds (sphere of 6 mm around the seed voxels specified above created using *‘3dUndump’*) to create maps of the mean Fisher’s *Z* transformed correlation for each seed voxel separately. The maps were then thresholded at *r* ≤ −.3 and *r* ≥ .3 after inverting Fisher’s *Z* transformation (*‘3dcalc -expr ‘tanh()’*) to create binary masks of first nearest neighbours (*‘3dClusterize’*) showing strong functional correlation with each seed region.

To compare these masks with established networks, parcellations from Yeo and colleagues^94^ were downloaded from https://surfer.nmr.mgh.harvard.edu/fswiki/CorticalParcellation_Yeo2011 and the Yeo2011_7Networks_MNI152_FreeSurferConformed1mm_LiberalMask.nii.gz file was resampled to the grid of our EPI data in MNI space (*‘3dresample’*) to then extract masks for visual, somatomotor and default network (*‘3dcalc’*). To specify similarity between the network and thresholded sFC masks, Dice coefficients were calculated (*‘3ddot -dodice’*). Additionally, the overlap between these networks and visual, auditory, and precuneus seed was quantified (*‘3dABoverlap’*).

#### Thresholding

All fMRI analyses are performed on whole-brain level applying a GM mask: during preprocessing (full pipeline), the GM mask based on FreeSurfer parcellation was transformed to MNI space and EPI resolution for each participant. These masks were averaged across the sample and thresholded at 0.5 so that a voxel is included to the group GM mask if it is included in the GM mask in at least 50% of the sample (*‘3dMean’* and *‘3dcalc’*). In total, the mask included 53,204 voxels as determined by *‘3dBrickStat’*.

To account for the multiple testing problem due to mass-univariate testing, stringent Bonferroni correction was applied using an initial *p* value threshold of α = 0.05 and dividing it by the number of voxels inside the mask multiplied by the number of tests carried out. In total, we here report eight tests on whole brain level (three One Sample *t*-tests, one Two Sample *t*-test, one ISC, and three sFC), leading to a Bonferroni corrected *p* value threshold of α = of 0.05/(8 * 53,204) = 0.0000001174724. Cluster extent thresholding was additionally carried out for this corrected *p* value based on the output of cluster size threshold simulations performed using *‘3dClustSim’* for first nearest neighbours clustering (faces of voxels touch) and a cluster threshold of α = of 0.05. This resulted in a threshold of two or three voxels for *t*-tests and ISC/sFC, respectively, that was applied as cluster-defining threshold to the results.

## Data Records

The raw (f)MRI data was converted from dicom to NIfTI format and standardized according to Brain Imaging Data Structure (BIDS)^95^ using pyBIDSconv v.1.1.9 (https://github.com/DrMichaelLindner/pyBIDSconv) facilitating sharing and the usage of BIDS apps^96^. Participants actively consented to their anonymised data from this project being made available to the research data. Anatomical data (i.e., identifiable facial features) and other information that could be used to directly identify participants have been removed from all records. The MMC Dataset is considered anonymised in compliance with the General Data Protection Regulation (GDPR) as the data shared cannot be used to identify participants using reasonable means^97^. Anatomical scans were defaced using AFNI’s ‘*@afni_refacer_run -mode_deface*’ and only defaced anatomical images were uploaded. Functional volumes acquired during the task after the scanner was stopped were discarded. The resulting files are available from the OpenNeuro platform for sharing neuroimaging data at https://openneuro.org/datasets/ds004182^98^ under a Creative Commons CC0 licence. In addition to the raw data, preprocessed data for each participant is shared in the derivatives directory together with files necessary for nuisance regression. While we here only share defaced data, during preprocessing, raw anatomical data that has not been defaced yet was used.

### Participant responses

**Location** derivatives/MMC_[resp].csv

**Response [resp]** demographics, scores, raw_quest_data, raw_corsi_data, raw_nback_data, other_information, scan_subj_sum, experimental_data

**File format** comma-separated value

Participants’ responses to demographic questions, questionnaires, and performance in the working memory assessment as well as both tasks are available in comma-separated value (CSV) files. Demographic (MMC_demographics.csv), raw questionnaire (MMC_raw_quest_data.csv) and other score data (MMC_scores.csv), as well as other information (MMC_other_information.csv), are structured as one line per participant with questions and/or scores as columns. Explicit wordings and naming of variables can be found in the Supplementary Information together with explanatory information related to testing durations reported in MMC_other_information.csv. Participant scan summaries (MMC_scan_subj_sum.csv) contain descriptives of brain coverage, tSNR, and FD (one row per participant) averaged first within acquisitions and then within participants. Participants’ responses and reaction times in the magic trick watching and memory task (MMC_experimental_data.csv) are stored as one row per trial per participant and a description of variables can be found in Supplementary Table 3. Raw responses obtained in the working memory tasks (MMC_raw_corsi_data.csv, MMC_raw_nback_data.csv) are available as one row per trial per participant and a description of variable names and values is included in the Supplementary Information.

### Anatomical data

**Location** sub-<group><ID>/anat/sub-<group><ID>_rec-NORM_T1w.nii.gz

**Space [space]** orig, MNI152NLin2009cAsym

**Description [desc]** skullstripped, surfvol

**Atlas name [atlas]** DesikanKilliany, Destrieux

**Mask label [label]** GM, WM, VENT, brain

**File format** NIfTI, gzip-compressed; plain text

**Sequence protocol** sub-<group><ID>/anat/sub-<group><ID>_rec-NORM_T1w.json

The raw, defaced anatomical images (pre-normalise filter applied by scanner) are available as a compressed 3D image file, stored as sub-<group><ID>/anat/sub-<group><ID>_rec-NORM_T1w.nii.gz.

To enhance re-usability (see Usage Notes for details), preprocessed anatomical data are shared. The skull-stripped image created using *@SSwarper* is available in original and ICBM 2009c Nonlinear Asymmetric Template space as derivatives/sub<group><ID>/anat/sub-<group><ID>_space-[space]_desc-skullstripped_T1w.nii.gz together with the corresponding affine matrix (derivatives/sub<group><ID>/anat/sub-<group><ID>_aff12.1D) and incremental warp (derivatives/sub<group><ID>/anat/sub-<group><ID>_warp.nii.gz).

Output generated using *@SUMA_Make_Spec_FS* (defaced anatomical image, whole brain and tissue masks, as well as FreeSurfer discrete segmentations based on the Desikan-Killiany cortical atlas^69^ and the Destrieux cortical atlas^99,100^) are also available as derivatives/sub<group><ID>/anat/sub-<group><ID>_space-orig_desc-surfvol_T1w.nii.gz, derivatives/sub<group><ID>/anat/sub-<group><ID>_space-orig_label-[label]_mask.nii.gz, and derivatives/sub<group><ID>/anat/sub-<group><ID>_space-orig_desc-[atlas]_dseg.nii.gz, respectively.

### Functional data

**Location** sub-<group><ID>/func/sub-<group><ID>_task-[task]_run-[1–3]_bold.nii.gz

**Task name [task]** magictrickwatching, rest

**File format** NIfTI, gzip-compressed; tab-separated value

**Sequence protocol** sub-<group><ID>/func/sub-<group><ID>_task-[task]_run-[1–3]_bold.json

#### Event file

derivatives/eventfiles_magictrickwatching/sub-<group><ID>/sub-<group><ID>_task-magictrickwatching_run-[1–3]_events.tsv

**Tissue mask label [tissue]** epiGM, epiWM, epiVentThe raw fMRI data (volumes for task data already discarded) are available as individual timeseries files, stored as sub-<group><ID>/func/sub-<group><ID>_task-[task]_run-[1–3]_bold.nii.gz. To enhance re-usability (see Usage Notes for details), the fully pre-processed and denoised files are shared as derivatives/sub-<group><ID>/func/sub-<group><ID>_task-[task]_desc-fullpreproc_bold.nii.gz. Additionally, partially pre-processed files (distortion corrected, despiked, slice-timing/head-motion corrected, aligned to anatomy and template space) are uploaded as derivatives/sub-<group><ID>/func/sub-<group><ID>_task-[task]_run-[1–3]_desc-MNIaligned_bold.nii.gz together with slightly dilated brain mask in EPI resolution and template space where white matter and lateral ventricle were removed (derivatives/sub-<group><ID>/func/sub-<group><ID>_task-[task]_space-MNI152NLin2009cAsym_label-dilatedGM_mask.nii.gz).

### Field maps

**Location** sub-<group><ID>/fmap/sub-<group><ID>_[map].nii.gz

**Field map name** [map] magnitude1, magnitude2, phasediff

**File format** NIfTI, gzip-compressed

**Sequence protocol** sub-<group><ID>/fmap/sub-<group><ID>_[map].json

The field maps are available as sub-<group><ID>/fmap/sub-<group><ID>_[map].nii.gz.

### Nuisance regressors

**Location** derivatives/sub-<group><ID>/regressors/sub-<group><ID>_task-[task][_run[1–3]]_label-[estimate]_regressor.[1D; nii.gz]

**Task name [task]** magictrickwatching, rest

**Estimates [estimate]** mot, motdeman, motderiv, ventriclePC, outlierfrac, censorTRs, localWM

**File format** plain text; NIfTI, gzip-compressed

All nuisance regressors stem from the outputs of *afni_proc.py* (full preprocessing pipeline, see description above). They are provided as space-delimited text values where each row represents one volume concatenated across all runs for each task separately. Those estimates that are provided per run contain the data for the volumes of one run and zeros for the volumes of other runs. This allows them to be regressed out separately for each run.

The motion estimates show rotation (degree counterclockwise) in roll, pitch, and yaw and displacement (mm) in superior, left, and posterior direction. In addition to the motion parameters with respect to the base volume (derivatives/sub-<group><ID>/regressors/sub-<group><ID>_task-[task]_label-mot_regressor.1D), motion derivatives (derivatives/sub-<group><ID>/regressors/sub-<group><ID>_task-[task]_run[1–3]_label-motderiv_regressor.1D) and demeaned motion parameters (derivatives/sub-<group><ID>/regressors/sub-<group><ID>_task-[task]_run[1–3]_label-motdemean_regressor.1D) are also available for each run separately. The derivatives/sub-<group><ID>/regressors/sub-<group><ID>_task-[task]_run[1–3]_label-ventriclePC_regressor.1D files contain time course of the first three PCs of the lateral ventricle per run. Additionally, outlier fractions for each volume are provided (derivatives/sub-<group><ID>/regressors/sub-<group><ID>_task-[task]_label-outlierfrac_regressor.1D) and derivatives/sub-<group><ID>/regressors/sub-<group><ID>_task-[task]_label-censorTRs_regressor.1D shows which volumes were censored because motion or outlier fraction exceeded the limits specified. The voxelwise time course of local WM regressors created using fast ANATICOR^74^ is shared as derivatives/sub-<group><ID>/regressors/sub-<group><ID>_task-[task]_label-localWM_regressor.nii.gz.

### Quality control reports

**Location** derivatives/mriqc/sub-<group><ID>/func/sub-<group><ID>_task-[task][_run[1–3]]_bold.json; derivatives/mriqc/sub-<group><ID>/anat/sub-<group><ID>_rec-NORM_T1w.json;

**Task name [task]** magictrickwatching, rest

**File format** json

**Individual html reports** derivatives/mriqc/sub-<group><ID>_task-[task][_run[1–3]]_bold.html; derivatives/mriqc/sub-<group><ID>_rec-NORM_T1w.html;

**Modality [mod]** T1w, bold

**Group reports** derivatives/mriqc/group_[mod].[tsv/html]; derivatives/mriqc/markdown_MRIQC_output.html

Each directory contains json files with the IQMs corresponding to the modality. A complete description of the metrics used by MRIQC can be found at https://mriqc.readthedocs.io/en/stable/measures.html. Additionally, the root directory (derivatives/mriqc/) contains the html reports corresponding to each scan as well as the group reports generated by MRIQC together with the R markdown html containing the results of the exploratory analysis as well as the web API comparison.

## Technical Validation

### Stimuli and behavioural data

#### Manipulation check

The incentives- and/or curiosity-motivated learning phase implemented multiple manipulations/assessments. Firstly, curiosity was elicited within subjects by using magic tricks taken from the MagicCAT stimulus set^6^. Secondly, the availability of extrinsic incentives was manipulated between subjects using a four-alternative forced-choice pseudo task. Thirdly, a week later, incidental memory for the magic tricks was assessed. In the MMC Dataset, rather than asking participants to provide a solution to the magic tricks, participants were instead asked to provide an estimate of how many people could figure out the solution to the magic trick (i.e., a judgement of how easily the magic trick could be figured out). This decision was motivated by several considerations. Theoretically, identifying solutions to magic tricks can lead to the occurrence of any ‘Aha!’ experiences. Such ‘Aha!’ experiences can enhance the recall performance on its own merit^101^ and could have confounded the effects of interest. However, it is important to note that ‘Aha!’ experiences are found in studies presenting the same magic trick repeatedly (e.g., three times) and hardly any magic tricks are (attempted to be) solved after the first viewing^102,103^. To further reduce the effect that solution insights may have on the memory effects measured here, as well as to not encourage rehearsal processes by asking participants to recall their solutions outside the MRI scanner, we decided to not ask participants to *actively* generate solutions. In previous paradigms investigating the interaction between those three components (i.e., curiosity, memory, incentives)^32,57^, the incentives manipulation was made contingent on identifying a correct solution (i.e., if participants found the correct solution, they were paid). This means that receiving the incentives (i.e., identifying the correct solution) was inevitably related to the participants’ resolution of curiosity and uncertainty. As such, our design choices have further addressed this inherent confoundedness of other studies in the field.

While the estimate rating (i.e., how many people are able to find the solution to the magic trick) was not regarded as an outcome measure of interest, we also computed the within-person correlation between the estimate rating and curiosity rating. The average within-person correlation was negative, *r* = −.49. One interpretation is that the estimate rating reflects participants’ confidence in understanding the solution, and when participants understood the solution (this could have also caused an ‘Aha!’ moment^101^), they found the magic tricks less interesting. Another interpretation is that participants found non-surprising tricks boring and assumed that for such tricks it is easy for other people to understand the solution (regardless of whether they actually understood the solution or not). We do not necessarily see this negative correlation as confounding because confidence or surprise is the constituent part of the feeling of curiosity^104^. However, depending on the purpose, researchers may want to use estimate ratings in their analysis.

There is no empirical data available that could qualify the correctness of the estimate responses. The MagicCAT stimulus set^6^ also does not include any normative information that could be used to directly determine how accurate the participants’ estimate ratings were. However, the MagicCAT stimulus set contains ratings on ‘confidence in the solution’. This could be seen as a proxy because arguably if participants are more confident that they had figured out the solution, they might also estimate that a larger number of people would be able to identify the solution. The average estimate rating (across stimuli) obtained in the MMC Dataset was indeed highly correlated with the confidence ratings reported in the MagicCAT stimulus set, *r* = .67, *t*(34) = 5.193, *p* < .001. Similar results were observed when correlating curiosity ratings averaged across stimuli, *r* = .69, *t*(34) = 5.551, *p* < .001. Likewise, the MagicCAT stimulus set also reported moderate negative mean within-person correlations between ratings of ‘confidence in the solution’ and ‘curiosity in the solution’ (mean *r* = −.42).

To verify the effectiveness of the incentives manipulation, participants in the experimental group were asked at the end of the MRI session to indicate whether they invested effort to increase their bonus payments and how much bonus payments they expected to have earned. Moreover, participants were asked whether they believed that they would receive rewards depending on their performance in the magic trick watching task at the end of the memory test. Indeed, participants in the experimental group reported that they invested efforts (*M* = 5.16, *SD* = 1.03, *min* = 3, *max* = 7; 1 = ‘strongly disagree’, 7 = ‘strongly agree’), expected reward payments (*M* = £4.23, *SD* = £4.13, *min* = £0.00, *max* = £18.00), and believed in the incentives manipulation (*M* = 3.36, *SD* = 1.73, *min* = 1, *max* = 6; 1 = ‘definitely agree’, 6 = ‘definitely disagree’).

Another concern, especially related to the curiosity manipulation, was whether participants’ previous experience in producing magic tricks could bias the perception of the magic tricks. Overall, participants reported having very little experience in producing magic tricks (*M* = 1.5, *SD* = 0.81, *min* = 1, *max* = 4; 1 = ‘never’, 6 = ‘very frequently’) with 32 (or 64%) reporting to never have had any prior experience. The participants’ average curiosity rating did not correlate with their reported experience producing magic tricks, *r* = −.02, *t*(48) = .918, *p* = .363. This replicates the findings reported in the normative rating data of the MagicCAT stimulus collection whereby experience in performing magic tricks did not predict participants’ curiosity ratings^6^.

To check whether we successfully measured incidental memory encoding, participants were asked at the end of the memory test to indicate whether they were aware that their memory would be tested later and whether they tried to encode the magic tricks while watching them (6 = ‘definitely agree’, 1 = ‘definitely disagree’). Answers revealed that participants were largely unaware that their memory would be tested (*M* = 3.76, *SD* = 1.27, *min* = 1, *max* = 6), but still had some intention to encode them (*M* = 3.12, *SD* = 1.99, *min* = 1, *max* = 6). However, no significant correlation was observed between any measures of encoding and participants’ self-reported awareness that their memory would be tested (all |*r*| ≤ .16, all *p* ≥ .273) or participants’ self-reported intention to encode the magic tricks (all |*r*| ≤ .14, all *p* ≥ .344). Likewise, reviewing the answers the participants gave when asked about their hypothesis regarding the aim of the study at the end of the MRI session, however, showed that, while some participants suspected the study to be related to curiosity, motivation, and reward, no participant named encoding, memory, and learning as main objectives.

#### Timing

To use stimulus annotations as well as intersubject correlation approaches, synchronous timing is important. To demonstrate timing accuracy, intended and actual stimulus timings were compared. The resulting differences (in ms) are summarised in Table 2 separately for each group. Overall, timing was largely accurate (range of mean difference per trial component: [−0.144 ms; 62.32 ms]; range of absolute mean difference per trial component: [1.301 ms; 62.32 ms]). Welch Two Sample *t*-tests did not indicate any group-specific timing issues (all *p* ≥ .664).

**Table 2:** Descriptive Summary of Deviation (in ms) Between Programmed and Observed Duration of Different Trial Components Separately for Each Group.

Trial component	Control group	Experimental group	Group comparison
Stimulus display duration	62.32 (78.831) [36.063; 678.637]	61.966 (77.63) [35.42; 636.866]	*t*(1797.576) = 0.096, *p* = .924
Fixation after stimulus display	0.407 (2.467) [−5.917; 10.271]	0.444 (2.212) [−6.435; 7.894]	*t*(1777) = −0.339, *p* = .735
Fixation between ratings	−0.114 (3.974) [−6.17; 33.398]	−0.103 (3.766) [−5.599; 39.348]	*t*(1792.803) = −0.062, *p* = .951
Fixation after ratings	0.356 (2.2) [−6.848; 8.099]	0.311 (2.255) [−8.92; 8.091]	*t*(1796.918) = 0.435, *p* = .664

Values represent *M* (*SD*) [*min*; *max*]. Differences were averaged across all 1800 trials. Group differences were tested for significance using Welch Two Sample *t*-Tests.

Variations seen in the stimulus display duration are most likely caused by system-induced latencies in displaying the video clips frame by frame. Additionally, the timestamp of video onset was collected when the video was loaded rather than when the first frame was displayed. The observed minimum delay ≥ 35.42 ms supports the assumption that loading in the video caused some processing time, hence adding to the differences between intended and observed presentation times. However, they are relatively minor given the low temporal resolution of fMRI and a TR of 2 s and can be accounted for in analysis by either including the actual duration or by using the average display duration for each stimulus as described above.

#### Encoding performance

To ensure that the MMC Dataset can be used to study memory, encoding performance for each participant was calculated for each memory level and summarised in Table 3. Recall performance was comparable to recollection-based recognition and roughly around 36–42%. As expected, recall performance was higher on the lenient compared to the strict criteria and higher encoding performance was found in recognition regardless of confidence and the remembered criteria (53–62%). These results generally overlap with what was observed in the behavioural pilots^30,35^ and encoding is significantly above zero (in the context of recall and remembered criteria) or chance (i.e., 25% given a four-alternative forced choice recognition paradigm), respectively (all *t*(49) ≥ 18.871, all *p* < .001).

**Table 3:** Average Ratings (Summarised Within Participants) for all Dependent Variables, their Standard Deviation (SD) Over all Trials, and Results of their Variance Decomposition.

	subject-wise *M* (*SD*) [*min*; *max*]	*SD* (all trials)	Subject variance	Stimulus variance	Subject x stimulus variance
Curiosity	4.407 (0.838) [2.611; 6.972]	1.68	22.77%	6.71%	70.53%
Confidence	3.747 (0.639) [1.694; 4.944]	1.696	12.19%	19.95%	67.85%
Cued recall (strict)	0.368 (0.138) [0.056; 0.667]	0.482	6.34%	30.09%	63.57%
Cued recall (lenient)	0.413 (0.143) [0.111; 0.722]	0.493	6.52%	26.14%	67.35%
Recognition (all confidence level)	0.626 (0.114) [0.417; 0.889]	0.484	3.33%	16.49%	80.18%
Recognition (high confidence)	0.421 (0.149) [0.083; 0.75]	0.494	6.89%	17.08%	76.03%
Remembered (strict or high)	0.531 (0.147) [0.194; 0.833]	0.499	6.63%	22.45%	70.93%
Remembered (lenient or high)	0.553 (0.148) [0.222; 0.861]	0.497	6.78%	20.33%	72.9%

Curiosity (scale 1 to 7) and confidence (scale 1 to 6) were directly rated by the participants during the magic trick watching task and recognition memory test, respectively. Memory measurements were coded as zero or one and the numbers represent the fraction remembered (out of 36 trials) on different thresholds. Cued recall captures the coded performance in the cued recall block applying strict and lenient criteria. Recognition captures whether the correct alternative was selected in the four-alternative forced-choice recognition paradigm (regardless of the confidence rating). High confidence recognition reflects that the correct alternative was selected with a confidence rating above three. A magic trick was coded as remembered if it was recalled or recognised with high confidence^63^. Overall, the MMC Dataset has good encoding performances while avoiding floor and ceiling effects. Likewise, high participant x stimulus variance suggests that the data is suitable to investigate within-person variability.

To determine how encoding performance evolved throughout the scans, mixed-effects models were used to predict encoding at trial level using task block (dummy-coded using the first task block as reference) as predictors. No effects of task block were found on any measures of encoding performance (all |*z*| ≤ 1.555, all *p* ≥ .120), further validating the pseudo-randomisation strategy applied.

#### Variance decomposition

Similar to other studies^6,79^, we examined whether this dataset is suited to investigate within-person variability in curiosity, confidence, and memory encoding using mixed-effects models to decompose the data into participant variance, stimulus variance, and participant x stimulus variance. Table 3 reports *SD* (computed over all 1800 trials) of each dependent variable as well as the percentage of variance explained by participant, stimulus, and participant x stimulus variance, respectively. The effect of participant variance ranges from 3.33 to 22.77%. Participant variance explains larger proportions of variance in ratings of curiosity (22.77%) and confidence (12.19%) compared to variance in memory encoding (3.33–6.89%) suggesting that individual differences may be larger in ratings compared to encoding or that the former is more prone to response biases^105^ than the latter. While stimulus variance explains a low proportion of variance in curiosity (6.71%), the effects are more pronounced in confidence (19.95%) and memory encoding (16.49 - 30.09%). Across all dependent variables, participant x stimulus variance effects between 63.57 and 80.18% indicate that the dataset has sufficient within-person variability. These findings are consistent with what has been reported in the magic trick stimulus database^6^ and with another study investigating the impact of epistemic emotions on encoding^79^.

### Imaging data

#### MRI data quality assessments using MRIQC

Assessing data quality using MRIQC showed that the MMC Dataset is of high quality. The comparison to the crowd-sourced data from the web API^84^ indicated that the IQMs from functional and anatomical data are comparable with other datasets. Reviewing the group html report, outliers were observed in some IQMs. These are highlighted in an R Markdown file summarising the explorative analyses of the functional data IQMs together with the web API comparison for anatomical and functional data. This file is shared together with all other outputs generated by MRIQC. Due to a lack of a ‘gold standard’ of MRI data quality, however, we did not exclude any participants based on the image quality assessment reported here. Instead, all outputs and reports are shared together with the data to allow other researchers to use the data as they find appropriate.

As noted above, we used non-defaced data for preprocessing. However, to protect participants’ privacy, defaced data is shared. Previous research indicated that removing facial features from anatomical MRI data can influence the results of automated preprocessing and analysis pipelines for anatomical data^106^. More specifically, using three clinical datasets, it was shown that automated analysis pipelines are more likely to fail after the removal of facial features and that volumetric analyses were impacted. One possible concern with respect to the MMC Dataset might be that data with facial features were used for preprocessing, but data without facial features is shared and that the removal of facial features could affect the results. To address this, MRIQC, a fully automated pipeline to compute IQMs as outcome measures, was run using anatomical scans before and after facial features were removed. Contrary to what was previously reported^106^, the execution of the automated pipeline was not more likely to fail if facial features were removed and in both cases, no execution failures were observed. However, comparing the IQMs estimated on data with and without facial features using paired *t*-tests resulted in statistically significant differences surviving Bonferroni correction at α = 0.05/68 = 0.00074 in 19 out of 68 IQMs (or 27.9%, see the R Markdown file for details). IQMs quantifying the smoothness of the data (i.e., ‘fwhm_[avg/x/y/z]’) were larger after defacing, indicating a smoother image. While the white matter fraction of the intracranial volume (i.e., ‘icvs_wm’) was estimated as higher after defacing, the IQM indicates data quality if normally distributed. Shapiro-Wilk tests indicated that the IQM is normally distributed in both cases. Median and range of intensity non-uniformity (bias field; i.e., ‘inu_[med/range]’) were smaller in the defaced group. As such, the median values were closer to one, thereby indicating higher quality. Residual partial volume errors in grey and white matter (i.e., ‘rpve_[gm/wm]’) were larger in the defaced data (mean difference ≤ 0.3), where higher values indicate lower quality. SNR estimates (i.e., ‘snr_[csf/gm/wm/total)]’ were larger in the defaced data, indicating better quality. Summary statistics of the average intensities in tissue masks (i.e., ‘summary_csf_median’, ‘summary_wm_[mad/n/p95/stdv]’) were lower after defacing. Lastly, the overlap with the tissue probability map for the cerebrospinal fluid (i.e., ‘tpm_overlap_csf’) increased after defacing, indicating better spatial normalisation. Overall, this suggests that the removal of facial features as done for the purpose of data sharing did not negatively impact the execution of an exemplar automated analysis pipeline. While systematic differences in some measures were observed, the overall conclusion that the anatomical data is of high quality was not impacted. While we cannot conclude that these findings can generalise to other automated pipelines that researchers may wish to apply to analyse the MMC Dataset, we have shared all derivatives of the data used to obtain the results reported here (e.g., GM tissue masks) that can be used as reference to compare against.

Overall, the IQMs extracted from the functional data were stable over the course of the experiment (mean participant-wise *SD* summarised across all standardised IQMs 0.35, *SD* = 0.25, *min* = 0.08, *max* = 1.08). The LME analysis showed that there were no group effects in any of the 37 functional IQMs (all *p* > .150). However, main effects of task were found for the number of volumes (‘size_t’: estimate −77.41, *p* < .001 surviving Bonferroni-corrected threshold of α = 0.05/37 = 0.0014), and two descriptive background statistics (‘summary_bg_mad’: estimate −0.09, *p* < .001; ‘summary_bg_median’: estimate −0.07, *p* < .001; all other *p* > .02), all indicating smaller values in resting state vs task data.

#### Brain coverage

Overall, most of the brain was scanned for the majority of scans as indicated by on average 98.20% (*SD* = 2.75%) of number of slices in z direction included in the brain mask covered by the EPI mask. More specifically, 85.66% of scans had either zero (51.0%) or one (34.7%) slices missing. The mean number of missing slices was 0.80 slices (*SD* = 1.24 slices) when averaged across scans or 0.81 slices (*SD* = 1.01 slices) when averaging within participants first.

The mean number of missing slices numerically increased throughout the course of the experiment, however, both groups were equally affected by this (see Table 4). This can most likely be attributed to the fact that participants have moved in the breaks in between scans as slice positioning was copied from the first resting-state scan without repeating localiser and slice positioning ahead of each scan. There were, however, no variations in brain coverage between the groups (all *p* > .228).

**Table 4:** Missing Slices for each Scan Separately for each Group.

	Scan	Control group	Experimental group	Group comparison
1	Pre-learning rest	0.6 (0.76) [3]	0.44 (1.04) [5]	*t*(43.969) = 0.618, *p* = .539
2	Magic trick task block 1	0.52 (0.77) [3]	0.8 (1.22) [5]	*t*(40.418) = −0.968, *p* = .339
3	Magic trick task block 2	0.56 (0.65) [2]	0.96 (1.61) [5]	*t*(33.197) = −1.175, *p* = .249
4	Magic trick task block 3	0.76 (0.78) [3]	1.16 (1.43) [5]	*t*(37.025) = −1.226, *p* = .228
5	Post-learing rest	1.12 (1.67) [8]	1.12 (1.69) [7]	*t*(47.99) = 0, *p* = 1

The extents of the EPI mask in inferior-superior direction were compared with those of the FreeSurfer parcellation mask. The table shows the mean difference (*SD*) [*max*] in slices for each group and scan separately together with the results of the Welch Two Sample *t*-Test.

#### Head motion

**Fig. 4:**
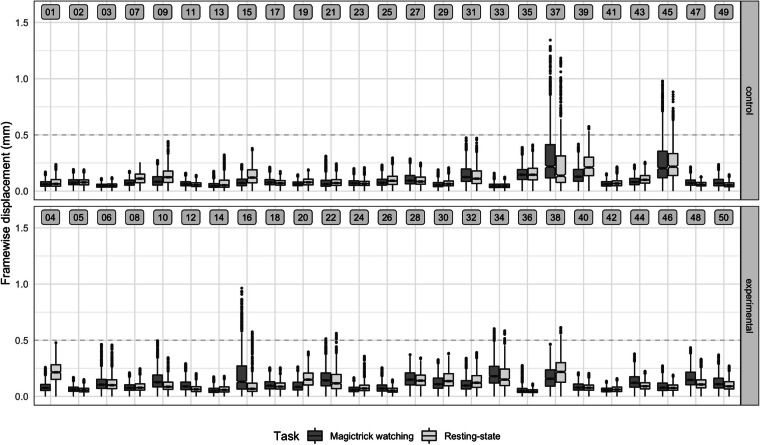
Framewise Displacement (FD, in mm) for each Participant. FD was calculated based on motion estimates from the minimal preprocessing pipeline. For each scanner run, FD was determined using the formula provided by Power and colleagues^85^ and concatenated within each task. Data for each task is shown as a notched boxplot where the left (dark gray) shows FD from the magic trick watching task (across all three blocks) and the right (light gray) the data from the resting-state scans (across both runs), respectively. To prevent scaling issues, outliers (values smaller 1.5*lower quantile or larger 1.5*upper quantile) were removed from the data before plotting. Two panels show data from the control group on the top and data from the experimental group on the bottom. Participant ID labels were added above the boxplots. FD was low in the MMC Dataset within each participant as indicated median values below the threshold of 0.5 mm (grey dashed line). Further, values were comparable during task and rest as well as for both groups.

For the MMC Dataset, mean FD across all scans as calculated by MRIQC was 0.13 mm. This is comparable to the mean FD of 0.16 mm computed based on the data from the MRIQC Web API^84^. Re-computing head motion based on the motion estimates from the full preprocessing pipeline led to similar results. Overall, head motion was low in the dataset as indicated by mean FD below 0.5 mm for all participants (mean of participant mean = 0.13 mm, *SD* = 0.06 mm, *min* = 0.06 mm, *max* = 0.33 mm) and is illustrated in Fig. 4 separately for each task.

#### Temporal signal-to-noise ratio (tSNR)

**Fig. 5:**
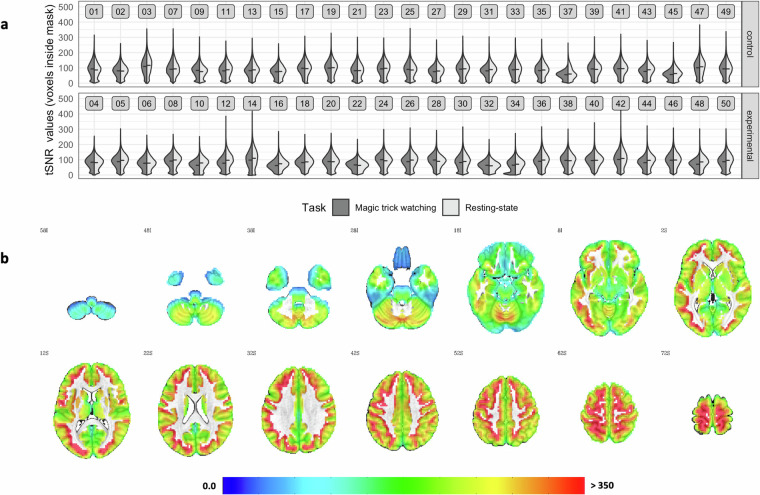
Temporal Signal-to-Noise Ratio (tSNR). (**a**) Values were extracted from the tSNR maps created as part of the minimal preprocessing pipeline using a dilated grey matter mask and concatenated within each task. Data is shown as a split violin plot where the left side (dark grey) of the plot shows estimates from the magic trick watching task (across all three blocks) and the right side (light grey) the data from the resting-state scans (across both runs), respectively. Horizontal cross bars indicate the median values. The panel structure is as in Fig. 4. Overall, minimally preprocessed timeseries show satisfactory tSNR values. Their distribution is comparable for task and rest and between groups. (**b**) To plot the distribution of voxel-wise tSNR values, the tSNR maps generated in the full preprocessing pipeline were averaged within each participant before computing the mean tSNR map shown. Overall, tSNR varies across the brain: cortical values are high while areas close to air-tissue boundaries show lower tSNR values.

tSNR values per scan as computed by MRIQC are comparable between MMC Dataset and Web API^84^ (mean median tSNR = 49.64 or 46.51, respectively). The minimally preprocessed dataset has a satisfactory tSNR (mean of participant mean = 85.48, *SD* = 11.64, *min* = 59.72, *max* = 111.59) which is comparable with previous dataset presenting naturalistic stimuli^87,90,107,108^. Additionally, Fig. 5a shows the distribution of tSNR values separately for task and rest whereas Fig. 5b shows the mean whole brain tSNR values surviving thresholding. As expected, all voxels show tSNR values significantly above zero, still there are variations in tSNR across different brain areas: while the values are especially high in lateral and superior cortical areas (shown in red), lower tSNR is found in e.g., inferior parts of the brain (shown in turquoise). The Two-Sample *t*-test revealed no significant differences in tSNR between the groups.

Upon inspection of the mean whole brain tSNR values, a left-right asymmetry in the tSNR values was noticed (for unthresholded results, please see https://neurovault.org/collections/13679/). Anatomy (including tissue composition) and sources of noise (e.g., physiological noise, scanner drift, and subject motion) may differ between the hemispheres and can hence result in left-right asymmetry in tSNR values^109–111^. While initially small, smoothing may have amplified inter-hemispheric differences, leading to the observed asymmetry^112^.

#### Intersubject correlation (ISC)

**Fig. 6:**
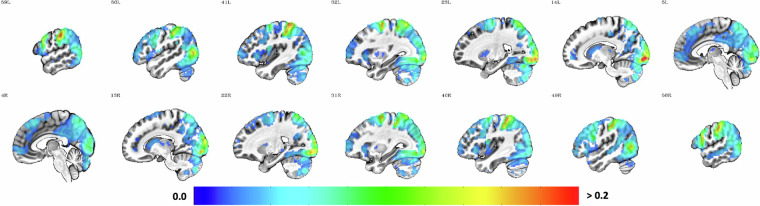
Intersubject Correlation Analysis. The map shows clusters surviving Bonferroni and cluster size extent thresholding. To avoid scaling issues, the top end of the colour bar was matched with *r* ≥ .2. Overall, the results validate the magic trick data by demonstrating synchronicity of areas across the brain with highest ISC values found in visual areas. ISC observed in other areas such as precuneus and temporo-parietal junction, regions putatively involved in various cognitive processes, as well as prefrontal cortices potentially highlight a plethora of cognitive processes and associated research questions that can be addressed with the MMC Dataset.

**Fig. 7:**
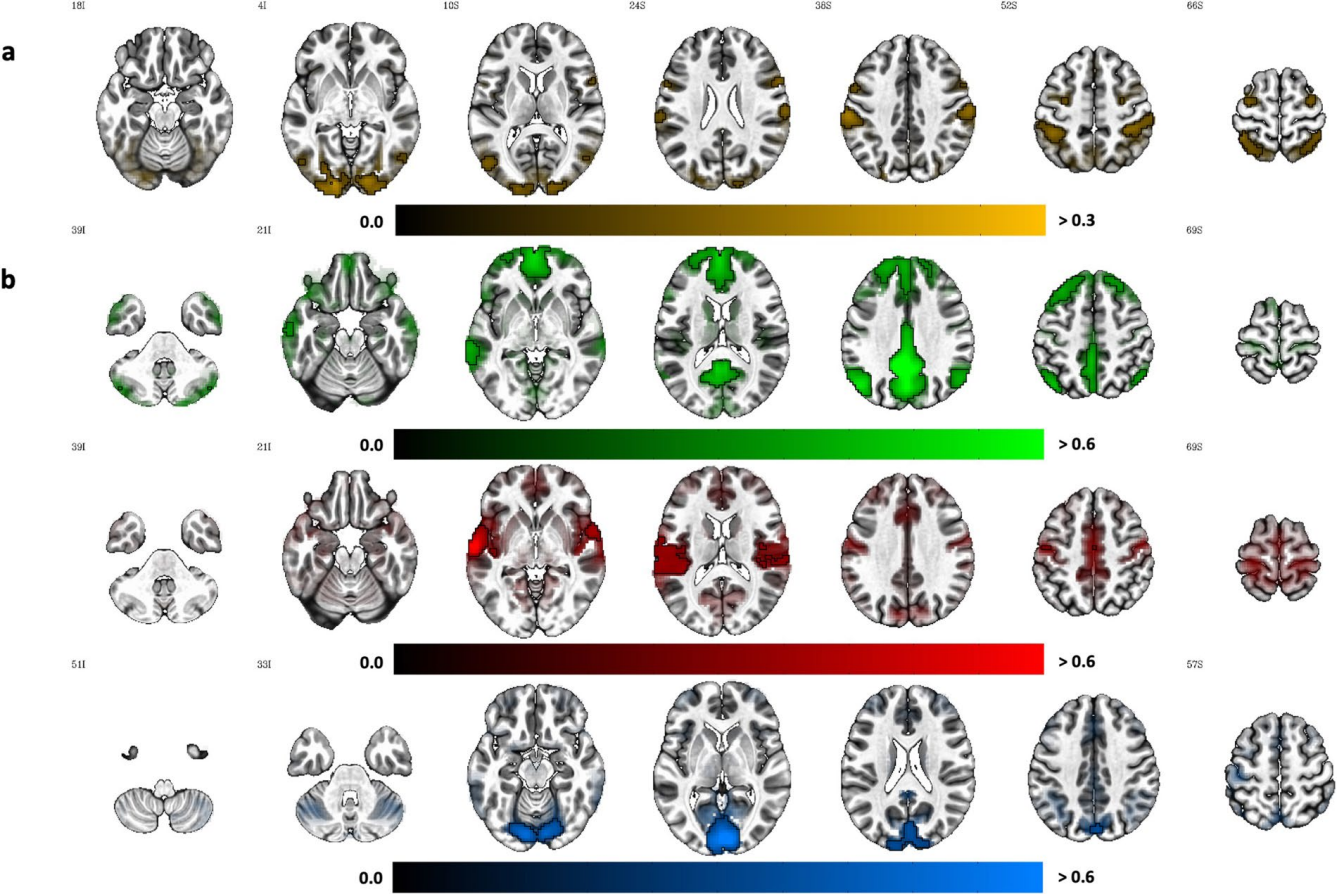
Summary of Basic Whole-Brain Data Validation Analyses. After correcting for multiple comparisons (using Bonferroni corrected and cluster-extent thresholding), the resulting maps were thresholded to only show voxels exceeding small (ISC) or moderate (sFC) correlation coefficients. The effect of thresholding was further softened by applying opacity information so that values closer to the threshold would be shown with higher opacity and a box was added around supra-threshold voxels only. (**a**) A threshold of *r* > .1 was applied to the ISC map plotted in Fig. 6 (shown in amber) highlighting high ISC in visual and sensory-motor areas. (**b**) To threshold the sFC maps in a similar manner as afni_proc.py’s automated QC scripts, *r* > .3 was used as the cut-off value. As expected, the precuneus seed (MNI 5 L, 49 P, 40 S; map shown in green) is correlated with other areas in the default mode network whereas the auditory seed (MNI 64 L, 12 P, 2 S) is predominantly functionally connected with other auditory and somatosensory areas (map shown in red). Likewise, the timeseries of the visual seed (MNI 4 R, 91 P, 3I) co-varies with activity in other parts of the visual cortex (map shown in blue).

To show that watching short magic tricks leads to similar intersubject synchronisation as reported when e.g., watching movies, ISC analysis were carried out. After Bonferroni-correction and cluster-extent thresholding, significant ISC was observed in large portions of the brain (see Fig. 6). More specifically, significant ISC was found in the cerebellum and visual cortices, spanning into the lateral and medial parietal cortex including the temporo-parietal junction, primary somatosensory cortices, and precuneus as well as the temporal lobe (e.g., middle temporal gyrus and fusiform gyrus). Frontally, clusters were located in (pre-)motor cortices, stretching into prefrontal areas including the precentral gyrus (frontal eye fields), lateral and anterior prefrontal cortex. Medially and subcortically, significant ISC was observed in the cingulate cortex and in the striatum and thalamus. While the maximum ISC (*r* = .25) is comparable to previous work^33,87^, most voxels had fairly low correlation values which is why additional thresholding at *r* > .1 was performed for plotting purposes (Fig. 7a). As expected, when presenting dynamic visual stimuli of moving body parts and objects, primary visual and association cortices as well as somatosensory and motor areas show high ISC values. In comparison, no ISC was observed in the primary auditory cortex which is consistent with the absence of sound in the video clips.

#### Seed-based resting-state functional connectivity (sFC)

Thresholded sFC maps using seeds located in the precuneus, auditory, and visual cortex were run to replicate data quality checks implemented in *‘afni_proc.py’* at single-subject level on group level. The thresholded sFC maps show distinct, non-overlapping cortical networks (all Dice coefficients of thresholded sFC maps created using different seeds ≤ .015) that are shown in Fig. 7b.

The precuneus seed (Fig. 7b, green) showed strong correlations with other voxels in the precuneus, posterior and anterior cingulate cortex, as well as dorso- and ventromedial prefrontal cortex (including frontal eye fields), angular gyrus and middle temporal lobe. Overall, it shows high similarity with the default network mask (Dice coefficient = .504) of which 90.625% of voxels are part of.

The sFC map created using the seed located in the auditory cortex (Fig. 7b, red) shows predominantly functional connectivity within the superior temporal gyrus and the frontoparietal operculum. Additionally, clusters in somatosensory and primary motor cortices and supplementary motor area were found. The sphere shares 84.849% of voxels with the somatomotor network defined by Yeo and colleagues^94^ with which its similarity is moderate (Dice coefficient = .219).

The seed located in the visual cortex (Fig. 7b, blue) exhibits strong correlations within the lateral and medial occipital lobe, cuneus, calcarine, and lingual gyrus. In total, 96.97% of voxels within the sphere are part of the visual network and the Dice coefficient of .294 shows satisfactory similarity.

## Usage Notes

Overall, the validation analyses showed that the MMC Dataset is of high quality. To facilitate reusability, we share the raw data as well as the output of the full preprocessing pipeline for functional and anatomical data (including tissue masks and skull-stripped images). We hope this will support researchers in navigating any added complexities with respect to implementing automated pipelines that can potentially arise due to the removal of facial features in the anatomical scans before sharing the data^106^. For researchers that wish to modify the preprocessing pipeline (e.g., by applying a different FWHM kernel or algorithm for smoothing), partially preprocessed data that is already in MNI space is shared together with all nuisance regressors used.

While naturalistic stimuli are often too rich to be captured in GLMs with predefined events modelled as regressors, specific features can be annotated and used as regressors to localise sensitive brain areas^26,113^ or time courses can be extracted in reverse correlation analyses to characterise contents in the movie leading to peaks and troughs in the time series of a voxel or ROI^27,114,115^. To assist such analysis approaches, we provide markers based on manual coding performed by the experimenters. However, these annotations can be extended in numerous directions. For instance, independent samples could rate the moment(s) of surprise for each magic trick, or a more objective approach could be developed. Antony and colleagues^116^ developed a model to characterise surprise in basketball games that were correlated with subjective ratings. Likewise, this dataset is also valuable in an emerging field referred to as the ‘science of magic’ where it can be used to study e.g., perception and other cognitive processes^1,2^. In a similar vein, given that the magic trick videos last multiple seconds, researchers may decide to exclude part of the data. While no eye-tracking data is available to aid such steps, eye gaze during naturalistic viewing on a TR-to-TR basis could be estimated using fMRI signal in the eye’s orbit^117^. The modified stimuli taken from the MagicCATs stimulus set used to generate this dataset were uploaded to OSF (https://osf.io/kaueg) and can be requested following the procedures outlined here (https://osf.io/ebpxq).

All in all, the MMC Dataset has the potential to foster our understanding of the neural underpinnings of curiosity- and incentives-motivated incidental learning as well as cognitive mechanisms related to attention or processing violations of predictions. However, some considerations are discussed below.

First, with respect to the MRI data acquisition, the study was conducted without multiband acceleration. While EPI multiband acquisition leads to a higher overall temporal resolution, this is not necessarily the case in regards to the detection of mesolimbic reward responses^118^. Further elevating such concerns, in recent fMRI studies, slice leakage artefacts were observed in protocols using multiband gradient echo EPI sequences (phase encoding direction P >> A, multiband factor 4). These artefacts were related to eye movements, reproducible, and generisable to other phase encoding directions (A >> P) as well as to multiband factor 2^119^. During naturalistic viewing, information is presented across the whole screen and the lack of fixations encourages free eye movements. Hence, to avoid the occurrence of inter-slice leakage and intra-slice aliasing, we opted for an acquisition protocol without multiband. In fact, the same protocol was deployed as used during the data acquisition of Lau and colleagues^18^ who also presented magic tricks inside the MRI scanner taken from the same database.

Second, it should be acknowledged that presenting naturalistic stimuli inside the MRI scanner is only an approximation to everyday life^26,120^. Although naturalistic stimuli share features with real-life situations where items are not perceived in isolation and detached from context but embedded in a dynamic, multisensory stream of fore- and background, passive naturalistic viewing paradigms cannot fully capture real-life, complex brain processes. This is amplified by restrictions of e.g., head movements and difficulties to understand the sound due to scanner noise.

Third, we used resting state scanning without any activities instead of active control tasks (e.g., performing arithmetics^121^ or movie watching^122^). This may raise a concern that our design is suceptible to mind wandering and intentional or spontaneous rumination/rehearsal during the post-learning rest phases. The decision against the use of active control tasks and movies was driven by the motivation to (a) increase comparability across studies implementing resting-state scans, which constitute the majority the field of consolidation research^59,123–128^, (b) ensure that the pre-learning resting state data could be re-used more broadly, possibly in combination with other datasets or using questionnaire data available, and (c) to circumvent the added analytic complexity related to active rest^121,129^. Relatedly, active control tasks are designed not to interfere with activity in brain areas and functions of interest^130^. As we cannot predict regions-of-interest of researchers who would re-use the MMC Dataset in the future, the benefits of rest phases rather than active control tasks outweighed the disadvantages in our considerations. Additionally, overlapping systems-level consolidation effects associated with e.g., motivated learning have been observed during active as well as passive rest^59,121,131,132^, hence reducing the likelihood that effects are solely driven by rumination/rehearsal. Note also that, when asked regarding the hypothesis about the study after MRI scanning, none of the participants suspected that the aim was to investigate memory. Likewise, the correlations between participants’ rating of their intention to encode and memory performance were small and non-significant. Therefore, we believe that the effect of rumination or rehearsal during the rest, if any, is minimal in the MMC Dataset.

Fourth, while the sample includes 50 participants, the data include a single between-subject factor (i.e., availability of incentives) and a moderate number of stimuli (i.e., 36 magic tricks), which may raise the concern regarding statistical power. It is important, however, to note that the primary usage of the current data is synchrony-based analysis^27,28^ (although the dataset is annotated to also support GLM-based analyses). Importantly, analysis approaches based on synchronisation can also incorporate behavioural covariates of interest (e.g., trait scores^75^, trial-level ratings^28,30^, encoding measures^30,133^). With this approach, the unit of analysis is based on the number of pairs of subjects (i.e., $\frac{1}{2}N\ast (N-1)$). While the statistical analysis corrects for data dependency due to the duplication of participants in pairs^77^, the effective sample size for this type of statistical analysis (e.g., mixed-effects modelling with crossed random effects) is generally higher than the original sample size. In fact, a previous statistical simulation using empirical fMRI data showed that the effective sample size was closer to the number of pairs, rather than the number of participants^134^. This point is relevant even when considering the number of stimuli and statistical power, because increased sample size generally has a bigger impact on statistical power than increasing the number of stimuli in fMRI analysis. We cannot provide a ‘general’ statistical power estimate with the MMC Dataset, because statistical power needs to be assessed on a case-by-case basis given research questions. However, we believe that this specific feature of the MMC Dataset addresses the issue of statistical power for many usage cases, although we should caution researchers that they should conduct a power analysis in advance to ensure that their planned analysis is well powered. Note that we chose the between-subject manipulation of incentive context on purpose so that researchers can answer substantial research questions regarding how the change in resting state brain activation is specifically associated with reward-motivated learning — if the incentive manipulation is within-subjects^59,131,132^, we could not compute the changes in resting-state brain activation specifically caused by the incentive contexts. This makes the MMC Dataset particularly valuable to study mechanisms of ‘adaptive consolidation’^31^.

To find a balance between rich, naturalistic stimuli on the one hand and experimental control, we decided to present magic trick video stimuli from a validated database that not only induce curiosity reliably, but also are as standardised as possible: for example, all magic tricks have the same dark background and viewing focus, the magicians wore similar clothing, and all videos are muted. While muted clips certainly circumvent the problems of scanner noise interfering with the auditory experience and language (and culture) dependency, the lack of multimodal stimulation also reduces the resemblance to real-world stimuli. On a broader level, one could argue that magic tricks themselves are not necessarily the most accurate presentation of real-world experiences and stimuli. However, on the continuum between highly controlled, laboratory stimuli and real-life, they are more naturalistic, dynamic, and complex than static stimuli like trivia questions presented on a screen. Taken together, the added ecological validity, richness, and complexity of the MMC Dataset combined with its high quality makes it a useful resource for researchers studying various features (e.g., curiosity, prediction errors) and cognitive functions (e.g., memory encoding and consolidation).

## Supplementary information

Supplementary Information

## Data Availability

All code is available in the Github repository https://github.com/stefaniemeliss/MMC_dataset. To reproduce all behavioural results, tables, and figures reported here, the R environment containing code and data is provided as a binder accessible here https://mybinder.org/v2/gh/stefaniemeliss/binder-MMC/master?urlpath=rstudio.
