# Supplementary material for: The magic, memory, and curiosity fMRI dataset of people viewing magic tricks
Source: Sci Data. 2024 Oct 1;11:1063. doi: 10.1038/s41597-024-03675-5 (PMC11445505; doi:10.1038/s41597-024-03675-5)
Supplement: Supplementary file 1 — Supplementary Information [file 41597_2024_3675_MOESM1_ESM.docx]

Supplementary material

Data Descriptor: Magic, Memory, and Curiosity - an fMRI dataset of people viewing magic tricks

Stefanie Meliss, Cristina Pascua Martin, Jeremy I Skipper, and Kou Murayama

Supplementary Information

[Pre-scanning online session 3](#_Toc133838710)

[Demographics 3](#_Toc133838711)

[MRI screening 5](#_Toc133838712)

[Questionnaires 6](#_Toc133838713)

[BIS BAS scale (Carver & White, 1994) 6](#_Toc133838714)

[Need for cognition (Cacioppo et al., 1984) 7](#_Toc133838715)

[Fear of failure (Spence & Helmreich, 1983) 8](#_Toc133838716)

[Approach and avoidance temperament (Elliot & Thrash, 2010) 8](#_Toc133838717)

[Melbourne Curiosity Inventory – Trait form (Naylor, 1981) 9](#_Toc133838718)

[Corsi 10](#_Toc133838719)

[Introduction 10](#_Toc133838720)

[Instructions 10](#_Toc133838721)

[2-back 10](#_Toc133838722)

[Introduction 10](#_Toc133838723)

[Instructions 10](#_Toc133838724)

[Feedback after practice 11](#_Toc133838725)

[Break 11](#_Toc133838726)

[Instructions for the magic trick watching task for the MRI session in the lab 12](#_Toc133838727)

[Instructions presented during practice 12](#_Toc133838728)

[Instructions presented during the MRI scanning 16](#_Toc133838729)

[Introduction 16](#_Toc133838730)

[Pre-learning rest 17](#_Toc133838731)

[Task 17](#_Toc133838732)

[Post-learning rest 23](#_Toc133838733)

[Questionnaire 24](#_Toc133838734)

[Instructions presented outside the scanner after scanning 26](#_Toc133838735)

[Melbourne Curiosity Inventory – State form (Naylor, 1981) 26](#_Toc133838736)

[Other post-experimental assessments 27](#_Toc133838737)

[Instructions presented in the memory test 28](#_Toc133838738)

[Introduction 28](#_Toc133838739)

[Recall 28](#_Toc133838740)

[Recognition 28](#_Toc133838741)

[Questionnaire 28](#_Toc133838742)

[Debrief 30](#_Toc133838743)

[Magic Trick Stimuli 31](#_Toc133838744)

[Table 1: Description of the magic tricks used as stimulus in the study 31](#_Toc133838745)

[Table 2: Description of video files used in this study 40](#_Toc133838746)

[Data Files 48](#_Toc133838747)

[MMC_raw_corsi_data.csv 48](#_Toc133838748)

[MMC_raw_nback_data.csv 48](#_Toc133838749)

[Table 3: Timing information included in MMC_other_information.csv 49](#_Toc133838750)

[Table 4: Variable dictionary for MMC_experimental_data.csv 51](#_Toc133838751)

[References 55](#_Toc133838752)

# Pre-scanning online session

The following questions, questionnaires, and instructions for the working memory (WM) assessment were presented to the participants during the pre scanning online session implemented using PsyToolkit (Stoet, 2010, 2017) version 2.5.3 (<https://www.psytoolkit.org/cgi-bin/psy2.5.3/survey?s=Y9a>). Information on how this information is referred to in the dataset is added in square brackets and italics.

1. Before you proceed, please confirm that you fulfil the inclusion criteria by ticking all boxes. If you do not fulfil a criteria, please do not proceed and get in touch with the researchers (email: memo.pcls.phd@gmail.com).
   *[inclusioncheck.[1-7] in MMC_raw_quest_data.csv]*

- I am aged between 18 and 45.
- I am healthy and do not suffer from any chronic illness or psychiatric disorder.
- I am not taking any psychoactive drugs.
- I am not currently nursing, pregnant, or intending to become pregnant.
- I do not have cognitive impairments.
- I have normal or corrected hearing and vision (using contact lenses).
- I speak English fluently.

## Demographics

1. How old are you? *[age in MMC_demographics.csv]*
2. What is your date of birth? (DD/MM/YYYY) *[DOB in MMC_demographics.csv]*
3. What sex has been assigned at birth? *[sex in MMC_demographics.csv]*

- Male
- Female

1. What is your gender? *[gender in MMC_demographics.csv]*

- Male
- Female
- I describe my gender differently

1. What is your ethnic origin? *[ethnicity in MMC_demographics.csv]*

- White - British
- Other White
- Asian or Asian British - Bangladeshi
- Asian or Asian British - Indian
- Asian or Asian British - Pakistani
- Asian or Asian British - Chinese
- Other Asian background
- Black or Black British - Carribbean
- Other Black background
- Mixed - White and Asian
- Mixed - White and Black African
- Mixed - White and Black Carribbean
- Other Mixed background
- Other Ethnic background
- Not known
- Information refused

1. Is English your first language? *[english in MMC_demographics.csv]*

- yes
- no
  1. At what age did you start learning English? *[ageEnglishAcquisition in MMC_demographics.csv]*

1. What is the highest level of education you have completed? *[education in MMC_demographics.csv]*

- Primary school
- GCSEs or equivalent
- A-Levels or equivalent
- University undergraduate program
- University post-graduate program
- Doctoral degree

1. How many years of education have you received, including primary school? (12 = HS diploma, 15 = Bachelor's degree) *[yearsOfEducation in MMC_demographics.csv]*
2. What is your employment status? *[employment in MMC_demographics.csv]*

- Unemployed
- Self-employed part-time
- Self-employed full-time
- Part-time employment within organisation/company
- Full-time employment within organisation/company
- Full-time student
- Part-time student
  1. Which subject do you study? *[studySubject in MMC_demographics.csv]*

1. Please specify your handedness. *[handedness in MMC_demographics.csv]*

- left
- right
- both

1. Do you have normal or corrected vision? *[vision in MMC_demographics.csv]*

- I have normal vision and do not need glasses or contact lenses.
- I have corrected vision and am using glasses or contact lenses.
  1. You mentioned before that you have corrected vision. We are doing eye tracking inside the scanner which means that we cannot provide you with MRI goggles as wearing them prevents us from being able to track your eyes. This means that you are only able to participate in the MRI study if your vision is corrected using contact lenses.
- I have corrected vision and CAN wear contact lenses for the MRI experiment.
- I have corrected vision and CANNOT wear contact lenses for the MRI experiment.

1. Please select the number that describes your health best. How would you describe your overall health, on a scale from 1 to 9 (1 = very poor health, 9 = excellent health)? *[health in MMC_demographics.csv]*
2. Are you currently under a doctor’s care for any of the following
   *[health_current.[1-4] in MMC_raw_quest_data.csv]*

- Heart disease (including coronary artery disease, angina, and arrhythmia)
- Vascular disease
- Diabetes
- I am not under a doctor's care for any of the items listed above.

1. Have you EVER been told by a doctor or other health professional that you had... (please tick any that applies)
   *[health_ever.[1-4] in MMC_raw_quest_data.csv]*

- Hypertension, also called high blood pressure
- Coronary heart disease
- A heart attack (also called myocardial infarction)
- Multiple sclerosis
- Parkinson’s disease
- Neuropathy
- Seizures
- Any kind of heart condition or heart disease
- A stroke
- Arthritis, rheumatoid arthritis, gout, lupus, or fibromyalgia
- Emphysema
- Cancer or a malignancy of any kind
- High cholesterol
- Diabetes or sugar diabetes
- Poor circulation in your legs
- Irregular heartbeats
- Congestive heart failure
- Asthma
- Osteoporosis or tendonitis
- Ulcers
- Varicose veins or haemorrhoids
- Narcolepsy
- Sleep Apnoea or other sleep disorder
- I have never been told by a doctor or other health professional that I have any of the items listed above.

## MRI screening

*[screening_MRI.[1-19] in MMC_raw_quest_data.csv]*

Thank you for providing some demographical information. As next step, we would like you to go through the MRI screening form. Please make sure to give these questions due considerations.

Please read the following questions CAREFULLY and provide answers. For a very small number of individuals, being scanned can endanger comfort, health or even life. The purpose of these questions is to make sure that you are not such a person.

You have the right to withdraw from the screening and subsequent scanning if you find the questions unacceptably intrusive. The information you provide will be treated as strictly confidential and will be held in secure conditions.

If you answer "yes" to any of the questions, please email memo.pcls.phd@gmail.com the specific details. If you answer "yes" to any of the questions 1 to 7, you **cannot** be scanned without risking your health and safety and hence have to be excluded as study participant. Please do not proceed in that case.

| 1. Have you been fitted with a pacemaker or artificial heart valve? | YES/NO |
| --- | --- |
| 1. Have you any active implants, such as cochlear, ocular, penile implant? | YES/NO |
| 1. Have you ever had any metal fragments in your eyes? | YES/NO |
| 1. Have you ever had any metal fragments, e.g. shrapnel in any other part of your body? | YES/NO |
| 1. Do you have any drug infusion pump installed? | YES/NO |
| 1. Do you have any stimulators for nerves, brain or bone installed? | YES/NO |
| 1. Is there any possibility that you might be pregnant? | YES/NO |
| 1. Have you ever been diagnosed with any form of heart disease or thermoregulatory problem? | YES/NO |
| 1. Have you any surgically implanted metal in any part of your body, other than dental fillings and crowns (e.g. joint replacement or bone re construction)? | YES/NO |
| 1. Have you ever had any surgery that might have involved metal implants? | YES/NO |
| 1. Have you ever suffered from epilepsy? | YES/NO |
| 1. Do you have an intrauterine contraceptive device (IUD) installed? | YES/NO |
| 1. Do you wear transdermal patches that contain metal? | YES/NO |
| 1. Do you wear a filling, crown, dental post (entirely within the tooth) associated with root canal treatment, retainer, bridge, or braces? | YES/NO |
| 1. Are you using coloured contact lenses? | YES/NO |
| 1. Do you wear a hearing aid? | YES/NO |
| 1. Do you have any body piercings that you cannot, or are unwilling to, remove? | YES/NO |
| 1. Do you have any tattoos or permanent make-up? | YES/NO |
| 1. Are you claustrophobic? | YES/NO |

## Questionnaires

Thank you for filling in the MRI screening form and providing some demographical information. Next we have some questionnaires. Please read all questions carefully and choose the answer that describes best how you feel. There are no correct or incorrect answers, so please just answer the questions as honestly as possible.

### BIS BAS scale (Carver & White, 1994)

*[BISBAS.[1-20] in MMC_raw_quest_data.csv; BISBAS_[score] in MMC_scores.csv with scores [score] = inhibition, rewardresponsiveness, drive, funseeking]*

Each item of this questionnaire is a statement that a person may either agree with or disagree with. For each item, indicate how much you agree or disagree with what the item says. Please respond to all the items; do not leave any blank. Choose only one response to each statement. Please be as accurate and honest as you can be. Respond to each item as if it were the only item. That is, don't worry about being "consistent" in your responses.

[options]

- very true for me
- somewhat true for me
- somewhat false for me
- very false for me

1. I worry about making mistakes.
2. Even if something bad is about to happen to me, I rarely experience fear or nervousness.
3. I go out of my way to get things I want.
4. When I'm doing well at something I love to keep at it.
5. I'm always willing to try something new if I think it will be fun.
6. It would excite me to win a contest.
7. When I get something I want, I feel excited and energised.
8. Criticism or scolding hurts me quite a bit.
9. When I want something I usually go all-out to get it.
10. I will often do things for no other reason than that they might be fun.
11. If I see a chance to get something I want I move on it right away.
12. I feel pretty worried or upset when I think or know somebody is angry at me.
13. When I see an opportunity for something I like I get excited right away.
14. I often act on the spur of the moment.
15. If I think something unpleasant is going to happen I usually get pretty "worked up."
16. When good things happen to me, it affects me strongly.
17. I feel worried when I think I have done poorly at something important.
18. I crave excitement and new sensations.
19. When I go after something I use a "no holds barred" approach.
20. I have very few fears compared to my friends.

### Need for cognition (Cacioppo et al., 1984)

*[NeedForCognition.[1-18] in MMC_raw_quest_data.csv, NeedForCogntion in MMC_scores.csv]*

Please describe the extent to which you agree with each statement using a 9-point scale ranging from very strong agreement to very strong disagreement.

[options]

- very strong agreement
- strong agreement
- moderate agreement
- slight agreement
- neither agreement nor disagreement
- slight disagreement
- moderate disagreement
- strong disagreement
- very strong disagreement

1. I would prefer complex to simple problems.
2. I like to have the responsibility of handling a situation that requires a lot of thinking.
3. Thinking is not my idea of fun.
4. I would rather do something that requires little thought than something that is sure to challenge my thinking abilities.
5. I try to anticipate and avoid situations where there is likely a chance I will have to think in depth about something.
6. I find satisfaction in deliberating hard and for long hours.
7. I only think as hard as I have to.
8. I prefer to think about small, daily projects to long-term ones.
9. I like tasks that require little thought once I’ve learned them.
10. The idea of relying on thought to make my way to the top appeals to me.
11. I really enjoy a task that involves coming up with new solutions to problems.
12. Learning new ways to think doesn’t excite me very much.
13. I prefer my life to be filled with puzzles that I must solve.
14. The notion of thinking abstractly is appealing to me.
15. I would prefer a task that is intellectual, difficult, and important to one that is somewhat important but does not require much thought.
16. I feel relief rather than satisfaction after completing a task that required a lot of mental effort.
17. It’s enough for me that something gets the job done; I don’t care how or why it works.
18. I usually end up deliberating about issues even when they do not affect me personally.

### Fear of failure (Spence & Helmreich, 1983)

*[FearOfFailure.[1-9] in MMC_raw_quest_data.csv, FearOfFailure in MMC_scores.csv]*

For each statement, please indicate your level of agreement or disagreement.

[options]

- strongly disagree
- disagree
- uncertain
- agree
- strongly agree

1. When I start doing poorly on a task, I feel like giving up.
2. If given a choice, I have a tendency to select a relatively easy task rather than risk failure.
3. When I fail at a task, I am even more certain that I lack the ability to perform the task.
4. I often find that I am well prepared for success on a task, but I do not perform the task well under pressure.
5. I tend to put forth a great deal of effort into a task, but I often know that this effort is of poor quality.
6. Sometimes I think it is better not to have tried at all, then to have tried and failed.
7. When I am tackling a challenging task, I find that I am reminded of my previous failures.
8. I often avoid a task because I am afraid that I will make mistakes.
9. I find that I can learn to perform a task very well, but I “crack” under the pressure of the situation and often do not perform anywhere close to my potential.

### Approach and avoidance temperament (Elliot & Thrash, 2010)

*[ApproachAndAvoidanceTemperament.[1-9] in MMC_raw_quest_data.csv, ApproachTemperament and AvoidanceTemperament in MMC_scores.csv]*

Please indicate how much you agree or disagree with each of the following statements by writing a number in the space provided. All of your responses are anonymous and confidential. Please select numbers according to the following scale:

1 = strongly disagree, 4 = neither agree nor disagree, 7 = strongly agree.

1. By nature, I am a very nervous person.
2. Thinking about the things I want really energizes me.
3. It doesn't take much to make me worry.
4. When I see an opportunity for something I like, I immediately get excited.
5. It doesn't take a lot to get me excited and motivated.
6. I feel anxiety and fear very deeply.
7. I react very strongly to bad experiences.
8. I'm always on the lookout for positive opportunities and experiences.
9. When it looks like something bad could happen, I have a strong urge to escape.
10. When good things happen to me, it affects me very strongly.
11. When I want something, I feel a strong desire to go after it.
12. It is easy for me to imagine bad things that might happen to me.

### Melbourne Curiosity Inventory – Trait form (Naylor, 1981)

*[TraitCuriosity.[1-18] in MMC_raw_quest_data.csv, TraitCuriosity in MMC_scores.csv]*

A number of statements which people have used to describe themselves are given below. Read each statement and then circle the appropriate number to the right of the statement to indicate how you generally feel.

There are no right or wrong answers. Do not spend too much time on any one statement but give the answer which seems to describe how you generally feel.

[options]

- almost never
- sometimes
- often
- almost always

- I think learning “about things” is interesting and exciting

- I am curious about things

- I enjoy taking things apart to “see what makes them tick’’

- I feel involved in what I do

- My spare time is filled with interesting activities

- I like to try to solve problems that puzzle me

- I enjoy exploring new places

- I feel active

- New situations capture my attention

- I feel inquisitive.

- I feel like asking questions about what is happening.

- The prospect of learning new things excites me.

- I feel like searching for answers.

- I like speculating about things.

- I like to experience new sensations.

- [I fee]l interested in things.

- I like to enquire about things I don’t understand.

- I feel like seeking things out.

- I want to probe deeply into things.

- I feel absorbed in things I do.

## Corsi

*[corsiSpan in MMC_scores.csv]*

### Introduction

Thank you for filling in the questionnaires.

As last part, we would like get a measurement of your cognitive abilities. First, we would like you to do the Corsi block tapping task.

In this implementation, we start with a sequence of 2 blocks. Once the sequence has been shown, you hear the word "go" (if you have your speakers on). You need to click with the mouse on the blocks in exactly the same order as shown before. When you are done, you click the green block "done". You get feedback (smiley face means you did it correct, or frowny face if you made a mistake). If you do it correctly, you go the the next higher number of blocks. If you do it wrong, you get once more chance. If you do it then wrong again, you get your score (the Corsi block span) and the task is over.

### Instructions

In this task, you need a mouse.

You will see 9 blocks.

Some will “light” up (yellow) in a sequence. Once you hear “go”, you need to click the same blocks in the same sequence. The sequences will increasingly get longer,

Press space bar when ready.

*[Corsi task]*

## 2-back

*[nBack_[score] in MMC_scores.csv with scores [score] = hits,* *misses_inclTooSlow, misses_exclTooSlow, correctrejections, falsealarms_inclTooSlow, falsealarms_exclTooSlow, hitrate, falsealarmrate, accurary]*

### Introduction

Thank you for doing the Corsi task. The last thing to do is a 2-back task.

There will be letters presented to you. You need to press the **m key** if the stimulus is the same as two trials ago, and the **n key** if not. The letters m and n are chosen for practical reasons (they can be easily remembered: m=memory, n=no).

You will start with a practice block followed by 4 task blocks in total. You will get feedback within the practice block, but not for the task blocks. You can take a break for as long as you like to between each of the blocks.

Once you are done with this task, please send an email to stefanie.meliss@pgr.reading.ac.uk with the completion code.

### Instructions

1. 2-back working memory task
   In this task, you will see a sequence of letters. Each letter is shown for a few seconds. You need to decide if you saw the same letter 2 trials ago, that is, this is a n=2-back task.
   If you saw the same letter 2 trials ago, please press **M** (M for Memory). If it was not a letter shown two trials ago, please press **N** (N for No).
   You need to press a button starting from the first letter presented.

   press the space bar for next info screen
2. 2-back working memory task
   We are going to start with a practice block with 20 trials. Afterwards, you will get some feedback on your performance.
   The actual task consists of 4 blocks with 20 trials each.
   Please feel free to take a break in between.

   press the space bar for next info screen
3. 2-back working memory task
   For example, these could be the letters and below the correct key press:

   A B L B T R H R I
   n n n **m** n n n **m** n

   This is actually very difficult! So you need some time to get good at it. When you respond correctly, you see the bars around the letter turning green and red, if wrong.

   press Q to start or arrow up to go back

### Feedback after practice

Percentage of missed 2-back items: xx % of all presented 2-backs

Percentage false alarms of 2-back item: xx % of all presented non 2-backs

Press space to finish practice and start first task block.

There will be no more red and green feedback.

The instructions will be shown once more.

### Break

2-back working memory task
Please take a break.

You need to decide if you saw the same letter 2 trials ago. If so, please press M (M for Memory). If it was not a letter shown 2 trials ago, please press N (N for no).

press Q for the next block

*[2-back task]*

# Instructions for the magic trick watching task for the MRI session in the lab

Different sets of instructions were presented to the participant during (1) practice, (2) while they were inside the MRI scanner, and (3) an assessment outside the MRI scanner. The instructions during practice and inside the MRI scanner were presented using PsychToolBox (Brainard, 1997) on a black screen using “Courier” font in white (unless indicated differently). The assessment outside the MRI scanner was implemented using PsyToolkit (Stoet, 2010, 2017) version 2.5.3 (<https://www.psytoolkit.org/cgi-bin/psy2.5.3/survey?s=JDPGx>). Instructions presented on the same screen are grouped together and in ascending order. Instructions were the same for both groups unless indicated differently. Text in square brackets was added to enhance readability, but not presented to the participant. They also contain information on additional communication with participants using the intercom from the scanner control room or when sequences where started.

## Instructions presented during practice

1. 'Hello and welcome!

   Thank you very much
   for participating in the experiment.'


   '(press any key to continue)'
2. 'We are going to practice
   the actual task.

   Do you feel ready?

   We will show you the instructions.'


   '(press any key to continue)'
3. 'In this experiment you will be presented
   with a series of magic tricks.
   The videos are without audio.

   Your task is to carefully watch the videos
   and try to figure out what has happened.'


   '(press any key to continue)'
4. 'Before the start of each magic trick
   you will see a fixation point.'


   '(press any key to continue)'
5. 'Afterwards, you are asked to give
   an estimate of how many people (out of 100)
   are able to correctly
   figure out the solution to the trick.

   Possible answers are the following:

   0 - 10 people
   11 - 20 people
   21 - 30 people
   31 or more people'


   '(press any key to continue)'
6. 'In addition to that, we would like you
   to rate how curious you were while watching
   the magic tricks on a scale

   from 1 (not curious at all) to 7 (very curious)'


   '(press any key to continue)'
7. 'For each of the answers,
   you have 6 seconds.'


   '(press any key to continue)'
8. 'To select the estimate you think
   is correct, you have to press
   the corresponding button on the button box.

   Your INDEX finger is lies on the blue button
   corresponding to the answer "0 to 10 people".
   Your MIDDLE finger is on the yellow button
   corresponding to "11 to 20 people".
   Your RING finger lies on the green button
   corresponding to "21 to 30 people"
   and your PINKIE lies on the red button
   corresponding to "31 and more people".'


   '(press any key to continue)'
9. 'For the curiosity rating, you have
   to move the red number
   to the number representing your curiosity.

   To move it to the left,
   please use your index finger (blue button).

   To move it to the right,
   please use your middle finger (yellow button).

   To confirm your selection,
   please use your pinkie (red button).'


   '(press any key to continue)'
10. 'You will see both the answer and
    the rating screen to show you
    how it looks like.

    After you have indicated your answer and
    your rating, the coloured ink will turn white
    and you simply wait for the task to continue.'


    '(press any key to continue)'
11. 'This is how it is going to look like:'

    *[Example rating answer]*
    'How many people (out of 100)
    are able to correctly figure out the solution?'

    '0 - 10 11 - 20 21 - 30 31 or more
    [in blue in yellow in green in red]
    '(index finger) (middle finger) (ring finger) (pinkie)'


    '(press any key to continue)'
12. 'This is how it is going to look like:'

    *[Example rating curiosity]*
    'Please rate how CURIOUS
    you were while watching the trick.'

    '1 2 3 4 5 6 7'
    (not at all) (very)


    '(press any key to continue)'
13. 'Do you have any questions?

    Please just ask.'


    '(press any key to continue)'
14. 'You will see two magictricks
    during the practice.

    In the actual experiment,
    there will be 36 magic tricks.'


    '(press any key to continue)'
15. 'The practice starts NOW.

    You are asked to estimate
    how many people are able to correctly find
    the solution to the magic trick.'


    '(press any key to continue)'


    [practice]
16. 'The practice is over now.

    Do you have any questions?'


    '(press any key to EXIT)'

## Instructions presented during the MRI scanning

### Introduction

*[check in with participant via intercom to tell them to read and follow the instructions on the screen]*

1. 'Hello and welcome!

   Thank you very much
   for participating in the experiment.'


   '(press any key to continue)'
2. 'Do you feel comfortable?

   If you would like to, you can have
   a little wiggle to make
   yourself even more comfortable.

   For the scanning, it is very important
   that you do not move your head.

   So please try to find a position
   that is as convenient as possible.'


   '(press any key to continue)'
3. 'We are going to run
   a short localizer sequence.

   Please keep your head as still as possible
   and continue reading.'


   '(press any key to continue)'

   *[Localizer scan starts]*
4. 'With the next sequence,
   we are measuring your brain activity at rest.

   The scan will last for approximately 10 min.
   You will see a white screen.

   Please keep your eyes open
   and simply look at the white screen.
   You are allowed to blink as usual.

   Please try to NOT think about anything at all.'


   '(press any key to continue)'
5. 'Do you have any questions?

   Please just ask.'


   '(press any key to continue)'

   [check in with participant via intercom to repeat the instructions verbally and clarify any questions]
6. 'To remind you:

   Please keep your head as still as possible

   and do not cross your legs or your arms.'


   '(press any key to continue)'

### Pre-learning rest

1. 'The screen will turn white shortly.

   Please keep your eyes open
   and try not to think about anything.'


   'scanning is starting, waiting for trigger'

   *[white screen presented for 10 minutes; EPI sequence]*
2. 'EXPERIMENTER INPUT:
   continue or abort'

### Task

*[check in with participant via intercom and offer of a break]*

1. 'Do you feel okay?

   Next, we are going to do
   the actual experiment.

   Do you feel ready?

   We will show you the instructions again.

   It is going to be the same task
   we practised earlier.'


   '(press any key to continue)'
2. 'While you are reading the instructions,
   we are running the fieldmap.
   So please keep your head as still as possible.
   There will be a screen asking you
   to wait so that someone can talk to you.
   Please do so once you get there.'


   '(press any key to continue)'

   *[field map scan starts]*
3. 'In this experiment you will be presented
   with a series of magic tricks.
   Your task is to carefully watch the videos
   and try to figure out what has happened.'


   '(press any key to continue)'
4. 'Afterwards, you are asked to give
   an estimate of how many people (out of 100)
   are able to correctly
   figure out the solution to the trick.

   Possible answers are the following:
   0 - 10 people
   11 - 20 people
   21 - 30 people
   31 or more people'


   '(press any key to continue)'
5. 'In addition to that, we would like you
   to rate how curious you were while watching
   the magic tricks on a scale

   from 1 (not curious at all) to 7 (very curious)'


   '(press any key to continue)'
6. 'For each of the answers,
   you have 6 seconds. '


   '(press any key to continue)'
7. *[Incentive manipulation (presented in experimental group only)]*

   'We ask you to answer the question
   "how many people are able to find
   the solution?" and you can get'
   ' an additional 50% bonus payment on top
   of your payment for both tasks (GBP 30.00)
   if you answer all questions correctly.
   That means each correct answer
   is worth an additional GBP 0.80' [presented in green]^[[1]](#footnote-1)^

   'Please press GREEN key (ring finger)
   to confirm that you read this statement.'

- Block 1

1. 'Please wait.

   Someone will talk to you shortly.'


   '(press any key to continue)'

   *[check in with participant via intercom to repeat the instructions and reward manipulation in experimental group only verbally and clarify any questions]*
2. 'In total, you will see 36 magic tricks.
   These will be presented in 3 blocks.

   There will be two breaks in between
   so that you can rest and relax.

   Please try not to move at all
   while you do the task.'


   '(press any key to continue)'
3. 'The experiment is ready to START.

   You are asked to estimate
   how many people are able to correctly find
   the solution to the magic trick.'


   '(press any key to continue)'
4. 'To remind you:

   Please keep your head as still as possible

   and do not cross your legs or your arms.'


   '(press any key to continue)'
5. 'The fixation point will show up shortly.'


   'scanning is starting, waiting for trigger'

   *[12 trials of magic trick watching task; EPI sequence]*

- Block 2

1. 'Thank you,
   the first block of the task is finished.

   WELL DONE!'


   '(press any key to continue)'
2. 'Please wait.

   Someone will talk to you shortly.'


   '(press any key to continue)'

   *[check in with participant via intercom and offer of a break]*
3. 'Take a break for as long as you need to.

   The next part of the experiment
   will start as soon as you are ready.

    The task is going to be
   the same as in the previous block.'


   '(press any key to continue)'
4. 'Do you have any questions?

   Please just ask.'


   '(press any key to continue)'
5. 'The experiment is ready to CONTINUE.

   You are again asked to estimate
   how many people are able to correctly
   find the solution to the magic trick.'

   *[Reward manipulation (presented in experimental group only)]*
   '[You can get] an additional 50% bonus payment on top
   of your payment for both tasks (GBP 30.00)
   if you answer all questions correctly.
   That means each correct answer
   is worth an additional GBP 0.80' [presented in green]

   'Please press GREEN key (ring finger)
   to confirm that you read this statement.'

   *[presented in control group only]*
   '(press any key to continue)'
6. 'To remind you:

   Please keep your head as still as possible

   and do not cross your legs or your arms.'


   '(press any key to continue)'

   *[check in with participant via intercom to repeat the instructions and reward manipulation in experimental group only verbally and clarify any questions]*
7. 'The fixation point will show up shortly.'


   'scanning is starting, waiting for trigger'

   *[12 trials of magic trick watching task; EPI sequence]*

- Block 3

1. 'Thank you,
   the second block of the task is finished.

   WELL DONE!'


   '(press any key to continue)'
2. 'Please wait.

   Someone will talk to you shortly.'


   '(press any key to continue)'

   *[check in with participant via intercom and offer of a break]*
3. 'Take a break for as long as you need to.

   The next part of the experiment
   will start as soon as you are ready.

    The task is going to be
   the same as in the previous block.'


   '(press any key to continue)'
4. 'Do you have any questions?

   Please just ask.'


   '(press any key to continue)'
5. 'The experiment is ready to CONTINUE.

   You are again asked to estimate
   how many people are able to correctly
   find the solution to the magic trick.'

   *[Reward manipulation (presented in experimental group only)]*
   '[You can get] an additional 50% bonus payment on top
   of your payment for both tasks (GBP 30.00)
   if you answer all questions correctly.
   That means each correct answer
   is worth an additional GBP 0.80' [presented in green]

   'Please press GREEN key (ring finger)
   to confirm that you read this statement.'

   *[presented in control group only]*
   '(press any key to continue)'
6. 'To remind you:

   Please keep your head as still as possible

   and do not cross your legs or your arms.'


   '(press any key to continue)'

   *[check in with participant via intercom to repeat the instructions and reward manipulation in experimental group only verbally and clarify any questions]*
7. 'The fixation point will show up shortly.'


   'scanning is starting, waiting for trigger'

   *[12 trials of magic trick watching task; EPI sequence]*

### Post-learning rest

1. 'The task is done, GOOD JOB!

   Thank you very much for completing it.'


   '(press any key to continue)'
2. 'Please wait.

   Someone will talk to you shortly.'


   '(press any key to continue) '

*[check in with participant via intercom and offer of a break]*

1. 'With the next sequence,
   we are measuring your brain activity at rest.

   The scan will last for approximately 10 min.
   You will see a white screen.

   Please keep your eyes open
   and simply look at the white screen.
   You are allowed to blink as usual.

   Please try to NOT think about anything at all.'


   '(press any key to continue)'
2. 'Do you have any questions?

   Please just ask.'


   '(press any key to continue)'

   *[check in with participant via intercom to repeat the instructions verbally and clarify any questions]*
3. 'To remind you:

   Please keep your head as still as possible

   and do not cross your legs or your arms.'


   '(press any key to continue)'
4. 'The screen will turn white shortly.

   Please keep your eyes open
   and try not to think about anything.'


   'scanning is starting, waiting for trigger'


   *[white screen presented for 10 minutes, EPI sequence]*
5. 'EXPERIMENTER INPUT:
   continue or abort'

### Questionnaire

1. 'Thank you.

   We are nearly done.'


   '(press any key to continue)'

   *[check in with participant via intercom, offer of a break and verbal explanation of how to do the questionnaire, start of T1 scan]*
2. 'We will start the last scan now.
   This is a structural image of your brain.
   That means you do not have to do
   anything at all.

   It will take approximately 6 minutes.'


   '(press any key to continue)'
3. 'To remind you:

   Please keep your head as still as possible

   and do not cross your legs or your arms.'


   '(press any key to continue)'
4. 'To prevent you from being too bored,
   we have prepared a questionnaire.

   This questionnaire is about
   your opinion of the experiment.'


   '(press any key to continue)'
5. 'Each question can be answered on a scale
   from 1 (definitely disagree) to 7 (definitely
   agree). Similar to the curiosity
   ratings, you have to move the red number
   to the number reflecting your opinion.

   To move it to the left,
   please use your index finger (blue button).

   To move it to the right,
   please use your middle finger (yellow button).

   To confirm your selection,
   please use your pinkie (red button).'


   '(press any key to continue)'
6. *[24-item questionnaire; each question presented on a single screen in random order; PostExpAssessment[1-24] in MMC_raw_quest_data.csv;
   IMI_[score] in MMC_scores.csv with scores [score] = intrinsicMotivation, interest, taskEngagement, boredom, effort, pressure;*

*compliance and ableToSee in MMC_other_information.csv]*

[intrinsic motivation (from Elliot & Harackiewicz, 1996): items 1-3
task engagement (from Elliot & Harackiewicz, 1996): items 4-6
interest (adopted from Wigfield & Eccles, 2000): items 7-9
boredom (adopted from Pekrun et al., 2002): items 9-12
effort (Ryan, 1982): items 13-17
pressure (Ryan, 1982): items 18-22]

*[answer scale]*1 2 3 4 5 6 7
definitely somehow slightly neither slightly somehow definitely
disagree disagree disagree disagree agree agree agree
 nor agree'

'It was fun to do the experiment. '

'It was boring to do the experiment.'

'It was enjoyable to do the experiment.'

'I was totally absorbed in the experiment.'

'I lost track of time.'

'I concentrated on the experiment.'

'The task was interesting.'

'I liked the experiment.'

'I found working on the task interesting.'

'The experiment bored me.'

'I found the experiment fairly dull.'

'I got bored.'

'I put a lot of effort into this.'

'I did not try very hard\nto do well at this activity.'

'I tried very hard on this activity.'

'It was important to me to do well at this task.'

'I did not put much energy into this.'

'I did not feel nervous at all while doing this.'

'I felt very tense while doing this activity.'

'I was very relaxed in doing this experiment.'

'I was anxious while working on this task.'

'I felt pressured while doing this task.'

'I tried to find out how many people will be able to find the solution.'

'I was able to see the magic tricks properly.'

## Instructions presented outside the scanner after scanning

### Melbourne Curiosity Inventory – State form (Naylor, 1981)

*[StateCuriosity[1-20] in MMC_raw_quest_data.csv, StateCuriosity in MMC_scores.csv]*

A number of statements which people have used to describe themselves are given below. Read each statement and then circle the appropriate number to the right of the statement to indicate how you generally feel.

There are no right or wrong answers. Do not spend too much time on any one statement but give the answer which seems to describe how you generally feel.

[options]

- not at all
- somewhat
- moderately so
- very much so

1. I want to know more.
2. I feel curious about what is happening.
3. I am feeling puzzled.
4. I want things to make sense.
5. I am intrigued by what is happening.
6. I want to probe deeply into things.
7. I am speculating about what is happening.
8. My curiosity is aroused.
9. I feel interested in things.
10. I feel inquisitive.
11. I feel like asking questions about what is happening.
12. Things feel incomplete.
13. I feel like seeking things out.
14. I feel like searching for answers.
15. I feel absorbed in what I am doing.
16. I want to explore possibilities.
17. My interest has been captured.
18. I feel involved in what I am doing.
19. I want more information.
20. I want to enquire further.

### Other post-experimental assessments

1. How many hours did you sleep last night? *[sleepLastNight in MMC_ other_information.csv]*
2. How many hours do you sleep on average each night? *[sleepAverage in MMC_ other_information.csv]*
3. Did you drink alcohol in the last 24 hours? *[alcohol in MMC_ other_information.csv]*
   1. How much alcohol did you drink during the last 24 hours? *[alcoholAmount in MMC_ other_information.csv]*
4. Please share some of your thoughts and opinions regarding the study with us. *[comment_task[1-3] in MMC_ other_information.csv]*

- Did you like the experiment? Why? Why not?
- What do you think is the hypothesis behind the experiment?
- Is there anything else you would like us to know?

*Comments added by the experimenters after data collection added as comment_exp in MMC_ other_information.csv*

[Items presented to experimental group only]

Choose one response that best describes how strongly each item applies to you:

1. I tried hard to increase my reward. *[rewardEffort in MMC_ other_information.csv]*

- Strongly disagree
- Somehow disagree
- Slightly disagree
- Neither agree nor disagree
- Slightly agree
- Somehow agree
- Strongly agree

1. How much additional bonus for correct answers do you expect? *[rewardExpectations in MMC_ other_information.csv]*

# Instructions presented in the memory test

## Introduction

Dear participant

Thank you very much for accepting the invitation to participate in the second part of the MAGMOT study.

During this part, you will be asked to try to remember the magic tricks you have seen inside the MRI scanner a week ago.

This includes a cued recall where we will present you still images of each of the 36 magic tricks and ask you to briefly describe what has happened in the magic trick. Depending on your answers in the recall tests, we will categories each magic trick you have seen in the first experiment as either remembered or forgotten. To enable us to do that, please try to be as distinct, specific and descriptive as possible when referring to the magic tricks.

Further, there will be a recognition test where you will see the same 36 still images and offered four choices to answer the question, "what happens in this magic trick?". You are asked to pick the correct answer and to indicate afterwards how confident you feel regarding your answer.

We estimate the duration of the task to be 45 min. You will receive additional course credit/monetary reward for your participation in this part of the study.

## Recall

In the following trials, we will present you still images of the 36 magic tricks you have seen in random order. Please write down what has happened in the trick cued by the picture.

Again, please try to write down your answer as descriptive as possible, so that we are able to judge whether you recalled a specific magic trick or not.

If you cannot recall what has happened in the magic trick cued by the still image, please insert "no recall".

*[36 cued recall trials]*

## Recognition

Thank you for completing the cued recall task.

Next, there will be a recognition memory test. You will see the same images as before in random order and again, you are asked to correctly recall what has happened in the magic trick. This time, however, you will be offered with four answers and we would kindly ask you to pick the correct one. Afterwards, please indicate how confident you are regarding your answer. That means if you are certain that this is the correct answer, your confidence will be high whereas it is low in case you are guessing.

*[36 recognition trials]*

## Questionnaire

Thank you for completing the recognition task.

You are nearly at the end of the experiment.

On the next page, there will be a short questionnaire. Afterwards, you will be debriefed and directed to the completion code.

Choose one response that best describes how strongly each item applies to you:

1. I slept between my participation in the first and second part of the study. *[sleepBeforeMemoryTest in MMC_ other_information.csv]*

- Yes
- No
  1. If yes, how many hours did you approximately sleep in total between your first and second participation? *[sleepHours in MMC_ other_information.csv]*

1. When watching the magic tricks, I was aware that my memory of them will be tested later. *[memoryTestKnown in MMC_ other_information.csv]*

- Definitely agree
- Somehow agree
- Slightly agree
- Slightly disagree
- Somehow disagree
- Definitely disagree

1. When watching the magic tricks, I tried to encode them. *[memoryIntention in MMC_ other_information.csv]*

- Definitely agree
- Somehow agree
- Slightly agree
- Slightly disagree
- Somehow disagree
- Definitely disagree

1. Please answer the following statement ONLY IF you have been offered **additional £0.80 bonus per correct answer**. If you have not been offered additional bonus, please select "not applicable."
   Did you believe that you would receive a bonus payment based on your performance? *[rewardBelief in MMC_ other_information.csv]*

- Definitely agree
- Somehow agree
- Slightly agree
- Slightly disagree
- Somehow disagree
- Definitely disagree
- Not applicable

1. I have experiences in producing magic tricks. *[magictrickExperience in MMC_ other_information.csv]*

- Very frequently
- Frequently
- Occasionally
- Rarely
- Very rarely
- Never

1. There were no problems with the internet connection while I participated in the experiment. *[connection in MMC_ other_information.csv]*

- Definitely agree
- Somehow agree
- Slightly agree
- Slightly disagree
- Somehow disagree
- Definitely disagree

1. Is there anything else you would like us to know? *[comment_memory in MMC_ other_information.csv]*

## Debrief

Dear participant,

Thank you for completing the task.

The purpose of this study was to find out how curiosity and the availability of reward can influence memory performances. We hypothesise that both, reward and high levels of curiosity will have enhancing effects and lead to the magic tricks being better encoded. We further investigate the neural underpinnings of this phenomena which we expect to be mirrored in the reward network, medial temporal lobe and mid brain.

We manipulated the availability of reward in the following ways: Some participants were not offered any reward for finding the correct estimate on how many people are able to find the solution, while other participants were offered an additional £0.80 for each correct estimate on how many people are able to find the solution to the magic trick.

We offered money to some of the participants to be able to look at the effect of the availability of monetary reward on memory. We asked you to rate your curiosity to determine how curiosity influences memory performances. We asked you to provide an estimate on how many people will be able to solve the magic trick to make sure that you are engaged in the task and pay attention to it. However, we never collected data on how many people are able to find the solution - therefore, there are no right or wrong answers to this question, and this was our cover story. We would like to apologise for any inconveniences caused by this. Out of curtesy, we pay the same bonus to all participants.

We would like to thank you again for participating in this experiment and supporting our research. If you have any additional questions, please do not hesitate to contact us.

Kind regards,

Stef Meliss (email: stefanie.meliss@pgr.reading.ac.uk) and Kou Murayama (email: k.murayama@reading.ac.uk)

# Magic Trick Stimuli

## Table 1: Description of the magic tricks used as stimulus in the study

| E/P? | Stim ID | Description | Recognition option 1 | Recognition option 2 | Recognition option 3 | Recognition option 4 |
| --- | --- | --- | --- | --- | --- | --- |
| E | H10 | Card that's split into four pieces is magically put back together. | The magician magically repairs split card. | The magician is able to split and put back together a card. | Without touching it, the magician is able to move the pieces of the cards further and further away from each other. | The magician places his hand over the card multiple times and each time a piece disappears. |
| E | H15 | Magician is able to balance a toothpick on a deck of cards but the volunteer cannot. | The magician balances a toothpick on a deck of cards. | The magician pierces the deck of cards with a toothpick, but afterwards there is no hole. | The magician lets a card picked by the volunteer appear in the card box. | When the magician starts to shake the card box, toothpicks start to fall out of the box and the cards have vanished. |
| E | H16 | Magician pours coke into glass from an empty can. | The magician pours coke into a glass from an empty can. | The magician is able to remove the dents from the can without touching it. | The magician pours coke into a glass and makes it disappear. | Although the can seems empty, the magician is able to pour sparkling water. |
| E | H17 | The magician uses a guillotine to slice breadsticks, then asks the volunteer to put her finger in. He slices the breadstick but not her finger. | The magician uses a guillotine to slice breadsticks, but not the volunteer's finger. | The magician rejoins bread sticks which the volunteer sliced with the guillotine. | The magician places the bread sticks in the bag, when the volunteer looks into the bag the bread sticks have transformed into a bun. | The volunteer tries to slice breadsticks with the guillotine, but cannot do it. |
| E | H19 | Magician places ball of paper in participants hand and sets it alight, revealing a red sponge ball. | The magician places paper in volunteer's hand and sets it alight, revealing a red sponge ball. | The magician lights matches without touching them. | The magician transforms matches into a lighter. | The magician presses a lit match into volunteer's hand without hurting her. |
| E | H35 | Volunteer signs card which the magician ends up pulling out of a Ziploc bag. | The volunteer signs a card which the magician subsequently pulls from a Ziploc bag. | While the magician is shuffling the deck of cards, all four aces start to appear in the Ziploc bag. | The magician puts all the cards into the Ziploc bag. After snapping their fingers the cards appear in the box and the bag is empty again. | The volunteer signs a card that is placed into the Ziploc bag, but then appears in the card box. |
| E | H36 | Screw and bolt unscrew itself. | The nut is placed on the middle of the bold by the volunteer and magically unscrews itself. | The magician shrinks the bolt, so that the nut does not stay on it any longer. | The magician places the nut in the middle of the bold and it magically screws itself. | The magician bends the bolt by hand. |
| E | H37 | Magician unscrews his finger and then shakes it back into place. | The magician unscrews his own finger and then shakes it back into place. | The magician claps his gloved hands, the gloves disappear leaving his hands bare. | The magician turns his gloves into mittens. | The magician detaches his thumb, but when he turns his hands, the thumb is reattached. |
| E | H7 | Coin appears everywhere around the salt case. | The magician lets a coin appear everywhere around the salt shaker. | The magician changes the salt to water. | The magician wraps the salt shaker in the cloth and makes it disappear. | The salt shaker turns into a bottle. |
| E | K10 | Safety pins magically interlock without breaking the seal. | The safety pins magically interlock without breaking the seal. | The safety pins magically double in size. | Although there are two safety pins at the beginning, they are magically melt together. | The magician uses the safety pins to create a little star. |
| E | K16 (s) | Volunteer selects and signs a card. Magician put it inside the deck and the signed card appeared on the top repeatedly. | The volunteer selects and signs a card that is placed in the middle of the deck, but then appears on the top of it. | The magician shuffles the cards and places them in front of him. When he taps the card with the pen, they spread out. | The magician covers the unsorted cards with a silk handkerchief. When the handkerchief is removed, each of the cards is marked. | The magician asks the volunteer to mark the cards using the pen. After he shuffles them, the volunteer's writing has disappeared from all of the cards. |
| E | K18 | Pack of cards comes out of a piece of paper. | A pack of cards appears from a single piece of paper. | The magician folds the sheet of paper smaller and smaller until it transforms into a pack of cards. | The magician splays out the cards more and more until they resemble bunting. | The magician turns the sheet of paper which now shows a bouquet of flowers. |
| E | K19 (l) | balls in a cup warps and turns into a toy rabbit. | The balls magically change their location and become a toy rabbit. | The print on the mug changes from a black Q10 to a red B2. | The red balls are placed under the mug and merge to become a form a larger ball. | The magician covers the mug and the red balls with a handkerchief. When he removes it, there is a tea pot with a red lid. |
| E | K2 | The sweets from a page in a children's story book are poured out onto the table from it. | Green and golden sweets fall out of the pages of the book. | The sweets shown on the pages disappear. | Gummi bears fall out of the book. | Micky and Mini Mouse from the pages of the book are poured out onto the table as little biscuits. |
| E | K21 (l) | A card flips with a short hand gesture. | The magician flips the middle card over with a slight gesture and then changes the colour of the back of the card from blue to red. | The magician reduces a full deck of cards to only three cards. | The magician places a card between two other cards and makes it disappear. | The magician transforms three cards into a full deck. |
| E | K24 | £10 note is transformed into a £2. | A £10 note is transformed into a £20 note. | The note is folded so small that it disappears. | The magician makes a note out of a flower. | The magician folds the ¬£10 note in a piece of paper and tears it. He reveals that the note is still intact afterwards. |
| E | K3 | A magician has a leaf on which two ladybirds appear on either side before magically disappearing. | The magician makes ladybirds appear and then disappear again. | The magician changes the shape of the leaf. | The magician takes the leaf and turns it into a box of flowers. | The magician changes the colour of the leaf several times. |
| E | K4 | The magician magically solves the rubix cube by spinning it in the air. | The magician solves the rubix cube by spinning it in the air. | The rubix cube levitates. | The rubix cube turns into a ball. | The magician shakes the rubix cube and it becomes all white. |
| E | S11 | Magician pushes a cigarette through a coin and then reveals that there is no hole. | The magician pushes a cigarette through a coin and then reveals that there is no hole. | The coin and the cigarette melt into another, so that the cigarette becomes metal. | The magician pushes a cigarette through a coin, but after he has pushed it through, he reveals that the cigarette is a £10 note. | The magician uses the coin to slice the cigarette, but it is still intact afterwards. |
| E | S12 | A hole is hole-punched into the corner of a card. The magician then moves the hole to a different corner and then reveals that it's actually a black spot on the card. | The magician punches a hole in the card. He then moves the hole from one corner to another before finally revealing that it is actually a black spot on the card. | The magician punches a hole into the card. This hole expands as the magician opens his fist. | The magician punches circular holes into the card, but after the card is turned, the punched holes have a square shape. | The magician punches a hole into the card, which then becomes a toy ladybird. |
| E | S15 | Magician places ring on the stem of a glass without breaking it. | The magician places a ring on the stem of the glass without breaking it. | The magician places the glass upside down on the ring and covers it with a piece of silk. When he removes it, the ring is placed on the stem. | The magician puts the ring into the glass and starts tossing it around until the ring is around the stem. | The magician removes the glass and puts a ring on the stem before reattaching it. |
| E | S18 | Magician pulls out 3 boxes with flowers in from a paper bag. | The magician pulls three boxes containing flowers from a paper bag. | The magician is able to hide his whole arm inside the blue paper bag. | The magician produces a rabbit out of the empty paper bag. | When the magician turns the bag inside-out the bag folds into a paper aeroplane. |
| E | S21 | Magician bends a spoon using fingers. | The magician detaches the head of a spoon by moving the handle up and down. | The magician detaches the head of a spoon by turning the handle clockwise and counter clockwise. | The magician bents the spoon only sing his pinkie as a force. | The magician covers the spoon with a handkerchief. When he removes it, the spoon is bent. |
| E | S25 | Handkerchief appears from bread. | The magician makes a silk handkerchief appear inside a bread roll. | The magician makes bread rolls appear in his hands. | The magician holds a bread roll in his left hand, which then appears in his right hand. | The magician makes a signed coin appear inside a bread roll. |
| E | S27 | Magician pours water into cup from bottle and then pours the water into another cup but when he tips it upside down, no water spills out. | The magician pours water into a cup from the bottle and then pours the water into another cup but when he tips it upside down, no water spills out. | The magician pours water into a cup from the bottle, but when he tips it upside down, no water spills out. | Although the magician pours water into both cups, the water bottle seems to refill itself magically whenever the magician snaps his fingers. | The magician pours water into one cup, but the other cup is magically refilled. |
| E | S30 | Magician pulls strings through a stick which makes the other string in the other stick move. | The magician pulls string through two sticks which appear to be connected. However he then reveals thw two sticks are not connected at all. | The magician is able to join the two separated parts of the stick. | Without any movement, the magician lets the strings in the sticks dance in little circles around him. | The magician cuts a piece of string in several places and then magically puts it back together again. |
| E | S31 | Magician places a pair of chopsticks into envelope and proceeds to crush the envelope and its contents into a paper ball. | The magician places chopsticks into an envelope, but is still able to crush the envelope and its contents into a paper ball. | The magician transforms the envelope into a ¬£10 note. | As the magician turns around the envelope, writing appears on it. | The magician folds the envelope multiple times until he reveals that it is a pair of chop sticks. |
| E | S9 | Magician turns a deck of cards into a blank deck. | The cards magically change their appearance. | The magician covers the cards with a silk handkerchief. After the silk is removed, the cards are blank. | The magician shows a card and then pulls the correct card from a shuffled deck. | The cards spread across the table into a circle without being touched. |
| E | Trick20 (s) | The magician shows a metal nut and rope. It doesnÕt matter how many times he secures the nut with the rope, it always scape. | The magician shows the volunteer metal nut and rope. Although he secures the nut with the rope, it repeatdely escapes. | The magician ties a knot around the volunteer's wrist using the rope. After snapping his finger, the nut is tied in the rope, too. | The volunteer holds the nut in her hands which are then tied with the rope. When the magician removes the rope, the nut is no longer in the volunteer's hand but appears in the magician's pocket. | The magician ties rope around the nut, but is not able to remove it afterwards as it seems that the nut has shrunk. |
| E | Trick28 | Magician links and unlinks several silver rings. | The magician links and unlinks several silver rings while they keep their shape. | Starting with small rings, the magician merges them so that there are less rings in total, but they are larger in size. | The magician firstly puts the rings around his arms, but after he turns around, the volunteer has them around their arms. | The magician links and unlinks several silver rings while the rings shape their appearance once they get linked. |
| E | Trick32 (l) | The magician makes a silver coin disappear and reappear several times. | The magician makes a silver coin disappear and reappear several times before it is transformed into a large coin. | The magician has a coin in each of his hands. They both end up in one hand. | The magician shows a silver and a copper coin. Every time he puts a coin in his pocket, the coin appears again in his hand. | The magician asks the volunteer for a coin. Then the coin magically bends inside the closed fist of the volunteer. |
| E | Trick37 | An elastic band visually travels from finger to finger, then it travels again even when the fingers are secured with another elastic band. | An elastic band visually travels from finger to finger. | The magician has an elastic band on his hand, which then magically ends up around the volunteer's hand. | The magician makes several star shapes using the two elastic bands and then produces a star shaped elastic band. | The magician has two elastic bands and tangles them up. They then become untwisted. |
| E | Trick38 | The magician closes his left fit around a silk, and makes it disappear. Then, he makes it appear from his right fist. | The magician closes one fist around a silk handkerchief and makes it disappear. Then, he makes it appear in his other hand. | The magician makes a little bird figure appear under the silk handkerchief. | The magician takes a coin from the volunteer and freezes it in the silk. | The magician closes his hand around a green silk handkerchief, but when he opens his hand, the silk is blue. |
| E | Trick4 | The magician puts a mobile phone inside a balloon with a simple gesture of his hands. | The magician puts the mobile phone inside the balloon with a simple gesture of his hands. | As the magician blows up the balloon, the phone increases in size. | The magician blows up an orange balloon, then the phone flashes and the balloon turns yellow. | As the balloon deflates, the phones starts moving across the surface without the magician touching it. |
| E | Trick6 (s) | The classic cup and ball routine using only 1 cup and an egg. | The magician puts a ball under the cup, which then turns into a larger ball and then becomes an egg. | The magician puts the ball under the cup which then turns into an egg and then becomes a chicklet. | The magician puts the ball under the cup and makes it disappear. | The magician places a red ball underneath the cup, but when he lifts it up, it is an orange balloon. |
| E | Trick7 (s) | The magician gets the volunteer to hold a foam ball which then doubles in to two. He repeats the trick and they then turn into three. | The magician gets the volunteer to hold a foam ball, which he then transforms into two balls. | The magician places two foam balls in volunteer's hands. The foam balls then start to jump up and down of their own accord. | The magician places two foam balls in volunteer's hand and sets them alight, revealing two paper balls. | Two sponge balls turn into a cube. |
| P | H4 (l) | Magician has four coins and two cards. He places the coins in each corner of the table and places two cards on top of the two top coins. He picks up one of the two remaining coins and makes it disappear from his hand. and then reveals it is now underneath one of the cards with the other coin. | | | | |
| P | K23 | Magician puts a hole through the money bill but then reveals that the hole disappears. | | | | |

*Note*. “E/P?” specifies whether the trick has been presented during the experiment (E) or during practice (P). “Stim ID” refers to the unique name of each magic trick. In cases where long (l) and short (s) versions of the same magic trick exists, the version used here is indicated. The description of the magic tricks stems from Ozono and colleagues (2021) where more information (e.g., name, credit, phenomena category, and materials) can be found and descriptions were taken there. The wording of the options for the recognition task has been piloted and tested in behavioural samples.

## Table 2: Description of video files used in this study

| Stim ID | Duration | Marker | Timing | Description | Notes |
| --- | --- | --- | --- | --- | --- |
| H10 | 36.84 (30.84) | Time stamp of cue image | 7.17 | H10_cue.png | |
|  |  | 1. moment of surprise | 18.2 | The magician fixes 3/4 bits of the broken card. | |
|  |  | 2. moment of surprise | 25.18 | The magician fixes remaining bit of card. | |
| H15 | 37.88 (31.88) | Time stamp of cue image | 7.15 | H15_cue.png | |
|  |  | 1. moment of surprise | 15.11 | The magician balances a toothpick on a deck of cards. | |
| H16 | 39.6 (33.6) | Time stamp of cue image | 7.2 | H16_cue.png | Main moment of surprise is the third moment of surprise |
|  |  | 1. moment of surprise | 19.22 | The crushed can is brought back to its original shape. | |
|  |  | 2. moment of surprise | 25.23 | Metal lid of can is closed. | |
|  |  | 3. moment of surprise | 29.23 | The magician pours a drink out of the can into the glass. | |
| H17 | 52.6 (46.6) | Time stamp of cue image | 6.24 | H17_cue.png | |
|  |  | 1. moment of surprise | 41.16 | The magician cuts the bread sticks with a guillotine whilst the volunteer has a finger in there as well. | |
|  |  | additional marker for 1. moment of surprise | 43.19 | The magician presents the volunteer's finger. | |
| H19 | 28.6 (22.6) | Time stamp of cue image | 9.21 | H19_cue.png | |
|  |  | 1. moment of surprise | 21.22 | The magician places ball of paper in volunteer's hand and sets it alight, revealing a red sponge ball. | |
| H35 | 43.64 (37.64) | Time stamp of cue image | 9.05 | H35_cue.png | |
|  |  | 1. moment of surprise | 40.02 | The volunteer signs a card which the magician subsequently pulls from a Ziploc bag. | |
| H36 | 42.4 (36.4) | Time stamp of cue image | 8.09 | H36_cue.png | |
|  |  | 1. moment of surprise | 29.03 | The nut is placed on the middle of the bold by the volunteer and magically unscrews itself. | |
|  |  | additional marker for 1. moment of surprise | 27.22 | The nut is unscrewing itself. | |
| H37 | 34.76 (28.76) | Time stamp of cue image | 7.17 | H37_cue.png | |
|  |  | 1. moment of surprise | 14.03 | The magician "breaks" his finger. | |
|  |  | 2. moment of surprise | 19.03 | The magician unscrews his finger. | |
|  |  | 3. moment of surprise | 24.03 | The magician screws his finger back on. | |
|  |  | 4. moment of surprise | 30.22 | The magician's finger appears normal again. | |
| H4 (l) | 63.04 (57.04) | Time stamp of cue image | 7 | H4_long_cue.png | |
|  |  | 1. moment of surprise | 25.03 | The coin disappears. | |
|  |  | 2. moment of surprise | 26.23 | The coin reappears under the card. | |
|  |  | 3. moment of surprise | 35.23 | The coin disappears again. | |
|  |  | 4. moment of surprise | 37.05 | The coin appears again. | |
|  |  | 5. moment of surprise | 54.22 | The coin disappears. | |
|  |  | 6. moment of surprise | 59.16 | All coins appear. | |
| H7 | 39.84 (33.84) | Time stamp of cue image | 7.08 | H7_cue.png |  |
|  |  | 1. moment of surprise | 15.2 | The coin appears in the magician's hand. | |
|  |  | 2. moment of surprise | 22.21 | The coin disappears from the magician's hand. | |
|  |  | 3. moment of surprise | 25.12 | The coin appears beneath the saltshaker. | |
|  |  | 4. moment of surprise | 31.22 | The coin disappears from magician's hand again. | |
|  |  | 5. moment of surprise | 35.23 | The coin appears on the top of the saltshaker. | |
| K10 | 29.4 (23.4) | Time stamp of cue image | 7.22 | K10_cue.png | |
|  |  | 1. moment of surprise | 9 | The safety pins magically interlock. | |
|  |  | 2. moment of surprise | 11.1 | The safety pins magically unlink. | |
|  |  | 3. moment of surprise | 14.05 | The safety pins magically interlock again. | |
|  |  | 4. moment of surprise | 16.11 | The safety pins magically unlink again. | |
|  |  | 5. moment of surprise | 17.05 | The safety pins magically interlock again. | |
| K16 (s) | 37.4 (31.4) | Time stamp of cue image | 7 | K16_short_cue.png | |
|  |  | 1. moment of surprise | 36.07 | The card that was marked by the volunteer appears on the top of the deck. | |
| K18 | 28.2 (22.2) | Time stamp of cue image | 12.2 | K18_cue.png | |
|  |  | 1. moment of surprise | 17.14 | The magician makes a deck of cards fall from the pictures. | |
| K19 (l) | 37.8 (31.8) | Time stamp of cue image | 9.11 | K19_long_cue.png | |
|  |  | 1. moment of surprise | 19.08 | A ball disappears from underneath the mug. | |
|  |  | 2. moment of surprise | 23.03 | The ball appears in volunteer's hand. | |
|  |  | 3. moment of surprise | 31.01 | Both balls disappear from magician's hand. | |
|  |  | 4. moment of surprise | 33.23 | A bunny appears when the mug is lifted. | |
| K2 | 33.2 (27.2) | Time stamp of cue image | 6.1 | K2_cue.png | Main moment of surprise is the first moment of surprise |
|  |  | 1. moment of surprise | 19.01 | The magician makes fall candy out a book. | |
|  |  | additional marker for 1. moment of surprise | 18.23 | The first piece of candy falls out of the book. | |
|  |  | 2. moment of surprise | 26.21 | The pictures of candy have disappeared from the book pages. | |
| K21 (l) | 49.76 (43.76) | Time stamp of cue image | 38.1 | K21_long_cue.png | |
|  |  | 1. moment of surprise | 22.08 | The magician flips the cards so that the one in the middle is facing up while the others are facing down. | |
|  |  | 2. moment of surprise | 42.08 | The magician changes the colour of one of the cards from blue to red. | |
| K23 | 41.4 (35.4) | Time stamp of cue image | 7 | K23_cue.png | |
|  |  | 1. moment of surprise | 36.15 | The magician puts a pen through the note and the hole disappears. | |
| K24 | 30.2 (24.2) | Time stamp of cue image | 8.15 | K24_cue.png | |
|  |  | 1. moment of surprise | 23.09 | A £10 note is transformed into a £20 note (partially visible) | |
|  |  | additional marker for 1. moment of surprise | 24.22 | Notes is almost completely unfolded and visible. | |
| K3 | 31.88 (25.88) | Time stamp of cue image | 8.14 | K3_cue.png |  |
|  |  | 1. moment of surprise | 22.02 | The magician makes ladybirds appear on a leaf. | |
|  |  | 2. moment of surprise | 27.15 | Ladybirds disappear. | |
| K4 | 37.16 (31.16) | Time stamp of cue image | 11.15 | K4_cue.png |  |
|  |  | 1. moment of surprise | 21.2 | The magician solves the rubix cube by spinning it in the air. | |
| S11 | 30.4 (24.4) | Time stamp of cue image | 6.13 | S11_cue.png | |
|  |  | 1. moment of surprise | 16.13 | The magician pushes a cigarette through a coin. | |
|  |  | 2. moment of surprise | 26.06 | It is revealed that the coin is not damaged. | |
| S12 | 47.88 (41.88) | Time stamp of cue image | 8.24 | S12_cue.png | |
|  |  | 1. moment of surprise | 24 | The hole is moved from the top left to bottom left corner. | |
|  |  | 2. moment of surprise | 32.03 | The magician moves the hole to the bottom left to bottom right corner. | |
|  |  | 3. moment of surprise | 44.19 | The hole turns into a black dot and the magician shakes it off. | |
| S15 | 37.96 (31.96) | Time stamp of cue image | 7.1 | S15_cue.png | |
|  |  | 1. moment of surprise | 34.08 | The magician places a ring on the stem of the glass without breaking it. | |
| S18 | 37.92 (31.92) | Time stamp of cue image | 14.16 | S18_cue.png | |
|  |  | 1. moment of surprise | 17.22 | The magician pulls out first box from the bag. | |
|  |  | 2. moment of surprise | 23.08 | The magician pulls out second box from the bag. | |
|  |  | 3. moment of surprise | 32.06 | The magician pulls out third box from the bag. | |
| S21 | 27.8 (21.8) | Time stamp of cue image | 7.15 | S21_cue.png | |
|  |  | 1. moment of surprise | 16.1 | The magician bents a spoon without applying force. It is more clear moment. | |
|  |  | additional marker for 1. moment of surprise | 13.2 | This is the first movement of finger and spoon. | |
|  |  | 2. moment of surprise | 24.05 | The spoon breaks into two pieces. | |
| S25 | 58.64 (52.64) | Time stamp of cue image | 29.07 | S25_cue.png | |
|  |  | 1. moment of surprise | 35.13 | A red cloth disappears from magicians hand. | |
|  |  | 2. moment of surprise | 48.23 | The cloth appears inside the bread roll. | |
| S27 | 37.56 (31.56) | Time stamp of cue image | 7.03 | S27_cue.png | |
|  |  | 1. moment of surprise | 33.04 | The magician pours the cup, but no water is coming out. | |
| S30 | 31.16 (25.16) | Time stamp of cue image | 23.01 | S30_cue.png | |
|  |  | 1. moment of surprise | 27.08 | The magician manipulates one string and with that also manipulates a second string that is not connected to the first one. | |
| S31 | 27 (21) | Time stamp of cue image | 18.01 | S31_cue.png | |
|  |  | 1. moment of surprise | 19.23 | The Magician can scrunch an envelope although chopsticks are inside it. | |
|  |  | additional marker for 1. moment of surprise | 20 | You can clearly see that the envelope is crushed. | |
| S9 | 38.44 (32.44) | Time stamp of cue image | 6.22 | S9_cue.png |  |
|  |  | 1. moment of surprise | 29.07 | The front side of the deck changes from colourful to white. | |
|  |  | additional marker for 1. moment of surprise | 30.11 | The whole deck is visible. | |
| Trick20 (s) | 32.12 (26.12) | Time stamp of cue image | 12.18 | Trick20_short_cue.png | |
|  |  | 1. moment of surprise | 30.01 | Although the magician secures the nut with a rope, it escapes. | |
| Trick28 | 46.44 (40.44) | Time stamp of cue image | 16.06 | Trick28_cue.png | |
|  |  | 1. moment of surprise | 17.06 | The magician links one ring to another. | |
|  |  | 2. moment of surprise | 25 | The magician unlinks the previously linked rings. | |
|  |  | 3. moment of surprise | 31 | The magician links two rings again. | |
|  |  | 4. moment of surprise | 36.03 | The magician links four rings together. | |
|  |  | 5. moment of surprise | 40.15 | The magician links more rings together. | |
|  |  | 6. moment of surprise | 45.06 | The magician displays all rings in a vertical line. | |
| Trick32 (l) | 52.92 (46.92) | Time stamp of cue image | 10.24 | Trick32_long_cue.png | |
|  |  | 1. moment of surprise | 15.22 | The coin disappears. | |
|  |  | 2. moment of surprise | 18.17 | The coin appears in the other hand. | |
|  |  | 3. moment of surprise | 24.24 | The coin changes hands again. | |
|  |  | 4. moment of surprise | 31.19 | The coin disappears again. | |
|  |  | 5. moment of surprise | 33.11 | The coin appears other hand. | |
|  |  | 6. moment of surprise | 42.15 | The coin appears in the magician's hand. | |
|  |  | 7. moment of surprise | 47.12 | The coin turns into a bigger coin. | |
| Trick37 | 52.28 (46.28) | Time stamp of cue image | 11.18 | Trick37_cue.png | |
|  |  | 1. moment of surprise | 24.2 | An elastic band visually travels from finger to finger. | |
|  |  | 2. moment of surprise | 47.15 | An elastic band visually travels from finger to finger, passing through the barrier. | |
| Trick38 | 33.4 (27.4) | Time stamp of cue image | 8.15 | Trick38_cue.png | |
|  |  | 1. moment of surprise | 21 | A green tissue disappears from the magician's hand. | |
|  |  | 2. moment of surprise | 26 | The tissue reappears in other hand. | |
| Trick4 | 53.8 (47.8) | Time stamp of cue image | 37.15 | Trick4_cue.png | |
|  |  | 1. moment of surprise | 40.23 | The phone appears inside the balloon. | |
|  |  | additional marker for 1. moment of surprise | 41.23 | Here it can be seen fully. | |
| Trick6 (s) | 43.64 (37.64) | Time stamp of cue image | 8.19 | Trick6_short_cue.png | |
|  |  | 1. moment of surprise | 21.14 | The ball appears under the cup. | |
|  |  | 2. moment of surprise | 32.18 | A bigger ball appears under the cup. | |
|  |  | 3. moment of surprise | 39.09 | An egg appears under the cup. | |
| Trick7 (s) | 26.6 (20.6) | Time stamp of cue image | 9.03 | Trick7_short_cue.png | Main moment of surprise is the second moment of surprise |
|  |  | 1. moment of surprise | 22.04 | A foam ball disappears from the magician's hand. | |
|  |  | 2. moment of surprise | 24.17 | The ball appears in volunteer's hand. | |
|  |  | additional marker for 2. moment of surprise | 25.07 | Two balls are clearly visible. | |

*Note*. For each magic trick, we included the duration (duration without the mock video), the marker and their associated timings, and descriptions for the moment(s) of surprise. The onsets of each of them have been added as markers in the PsychophysicsToolbox script and the experimental data files. All durations and timings are measured in seconds. As in the previous table, “Stim ID” refers to the unique name of each magic trick. In cases where long (l) and short (s) versions of the same magic trick exists, the version used here is indicated.

# Data Files

## MMC_raw_corsi_data.csv

| **ID** | **BIDS** | **max** | **span** | **response** | **condition** |
| --- | --- | --- | --- | --- | --- |
| 1 | sub-control001 | 2 | 2 | 1 | 298 |
| 1 | sub-control001 | 3 | 3 | 1 | 159 |
| 1 | sub-control001 | 4 | 4 | 1 | 7 |
| … | … | … | … | … | … |

max - highest corsi span thus far

span - number of items to remember

response - correct/incorrect

condition - random presentation of blocks on screen

## MMC_raw_nback_data.csv

| **ID** | **BIDS** | **block** | **response** | **key_pressed** | **rt_ms** | **condition** | **trial_number** | **current_letter** | **previous_letter** |
| --- | --- | --- | --- | --- | --- | --- | --- | --- | --- |
| 1 | sub-control001 | block1 | 1 | 2 | 895 | 3 | 1 | 2 | 5 |
| 1 | sub-control001 | block1 | 1 | 2 | 1457 | 1 | 2 | 7 | 2 |
| 1 | sub-control001 | block1 | 1 | 2 | 1547 | 2 | 3 | 7 | 7 |
| … | … | … | … | … | … | … | … | … | … |

block - Name of block (block[1:4] or training)

response - Participant’s response (1=correct, 2=wrong, 3=too slow)

key_pressed - which key was pressed (1 = ‘M’, 2 = ‘N’)

rt_ms - reaction time in milliseconds

condition - random number used for conditions (1=same as 2-back, 2-5 other letter)

trial_number - trial number (per block) [1:20]

current_letter - letter presented in current trial [1:15]

previous_letter - letter presented in previous trial [1:15]

## Table 3: Timing information included in MMC_other_information.csv

| Column Name | Explanation |
| --- | --- |
| daysBetweenPreAndExp | time difference between end of the pre scanning online session and the start of the magic trick watching task in days  *Note: For participant have negative numbers, indicating that the pre-scanning session was completed after the scanning. Two of them (sub-control001 and sub-control003) were part of the six initial pilot participants when the standard operating procedures were still being finalised. The other two (sub-control021 and sub-control035) initially completed the online assessment ahead of their scanning session, however, were directed to the end of the assessment before providing all necessary information. They were hence asked to complete the session again when this issue was noticed by the research team.* |
| daysBetweenExpAndMemory | time difference between end of magic trick watching task and start of memory test in days |
| durPre_min | duration pre scanning online session in minutes calculated as difference between start and end time stamp with minute precision provided by the PsyToolkit software |
| durPersInfo_min | amount of time in minutes participants needed to provide personal information (i.e., demographics, current and lifetime disease diagnoses, MRI safety criteria) in the pre scanning online session |
| durQuestionnaires_min | amount of time in minutes participants needed to complete questionnaires (i.e., BIS/BAS, Need For Cognition, Fear Of Failure, Approach And Avoidance Temperament, Trait Curiosity) in the pre scanning online session |
| durCorsi_min | amount of time in minutes participants viewed the Corsi introduction in the pre scanning online session |
| durNback_min | amount of time in minutes participants viewed the 2-back introduction in the pre scanning online session |
| durMemory_min | duration memory test in minutes |
| durRecall_min | duration recall block in minutes (i.e., time difference between start of first and end of last trial) |
| durRecognition_min | duration recognition block in minutes (i.e., time difference between start of first and end of last trial) |

| durScanning_min | duration scanning session (i.e., time difference in minutes between the time stamp collected at the end of the practice in minute precision and the time stamp collected at moment participant finished the task motivation inventory inside the scanner in minute precision)  *Note: For three participants (sub-experimental008, sub-experimental020, sub-experimental050), the time stamp for end of practice was not saved due to technical errors*  *.* |
| --- | --- |
| durMainExp_min | duration main experiment (i.e., magic trick watching task) in minutes measured from beginning of first trial in the first block to end of last trial in the third block.  *Note: The absolute time stamp of the end of the last trial within each block was recorded with minute precision, however, no absolute time stamp for the beginning of each block was recorded. However, the duration of each task block was recorded with millisecond precision. The start of the first task block was hence reconstructed by subtracting the duration of the first block from the time stamp of the end of the first block. This reconstructed time stamp (with second precision) was then used to calculate the duration of the task.* |
| dur_firstBlock_s | duration of first block of magic trick watching task in seconds |
| dur_firstBlock_min | duration of first block of magic trick watching task in minutes |
| dur_secondBlock_s | duration of second block of magic trick watching task in seconds |
| dur_secondBlock_min | duration of second block of magic trick watching task in minutes |
| dur_thirdBlock_s | duration of third block of magic trick watching task in seconds |
| dur_thirdBlock_min | duration of third block of magic trick watching task in minutes |
| dur_s | cumulated duration of all blocks of magic trick watching task in seconds |
| dur_min | cumulated duration of all blocks of magic trick watching task in minutes |

## Table 4: Variable dictionary for MMC_experimental_data.csv

| Variable | Explanation |
| --- | --- |
| ID | subject ID |
| BIDS | BIDS identifier |
| group | group: exp = experimental, cont = control |
| orderNumber | number of trial order task |
| block | task block |
| acq | acquisition within block |
| startBlock | time stamp (in secs) start of block |
| endBlock | time stamp (in secs) end of block |
| timingCorrection | half of the flip interval applied as timing correction (in secs) in PTB script |
| jitterVideo_trial | intended jitter interval (in secs) after magic trick display |
| jitterRating_trial | intended jitter interval (in secs) after curiosity rating |
| stimID | magic trick stimulus ID |
| vidFileName | file name of magic trick video |
| trial | trial number in magic trick task |
| tTrialStart | time stamp (in secs) start of trial |
| tTrialEnd | time stamp (in secs) end of trial |
| durationTrial | duration of trial (in secs) |
| fixationInitialDuration | duration of initial fixation at start of each block |
| displayVidOnset | time stamp (in secs) of magic trick onset; collected before the video was opened |
| displayVidOffset | time stamp (in secs) of magic trick offset; collected after the video was closed |
| displayVidDuration | observed display duration (in secs) of magic trick |
| displayBlankDuration | duration of blank presentation (in secs) between end of magic trick and onset of fixation |
| fixationPostVidOnset | time stamp (in secs) of onset of fixation after magic trick display |
| fixationPostVidDuration | duration (in secs) of display of fixation after magic trick display |
| displayAnswerOnset | time stamp (in secs) of onset of estimate rating |
| displayAnswerDuration | duration (in secs) of display of estimate rating |
| timeoutAnswer | response time window for estimate rating |
| responseAnswer | response given in estimate rating |
| timestampAnswer | time stamp (in secs) of response in estimate rating |
| timestampAnswerWhite | time stamp (in secs) of estimate rating being displayed in white ink |
| rtAnswer | response time for estimate rating |
| answer_tooSlow | 1 if time stamp response estimate > response time window for estimate rating - 3 * timing correction; else 0; subject sum score in other_information.csv ('answer_tooSlow') |
| fixationPostAnswerOnset | time stamp (in secs) of onset of fixation after estimate rating |
| fixationPostAnswerDuration | duration (in secs) of display of fixation after estimate rating |
| betweenRatingFixation | intended duration (in secs) for fixation after estimate rating |
| displayCuriosityOnset | time stamp (in secs) of onset of curiosity rating |
| displayCuriosityDuration | duration (in secs) of display of curiosity rating |
| timeoutCuriosity | response time window for curiosity rating |
| responseCuriosity | response given in curiosity rating |
| timestampCuriosity | time stamp (in secs) of response in curiosity rating |
| timestampCuriosityWhite | time stamp (in secs) of curiosity rating being displayed in white ink |
| rtCuriosity | response time for curiosity rating |
| startValueCuriosity | number that was highlighted in red at the beginning of the curiosity rating |
| clicksCuriosity | number of button presses to move the number to the left and right in the curiosity rating |
| fixationPostCuriosityOnset | time stamp (in secs) of onset of fixation after curiosity rating |
| fixationPostCuriosityDuration | duration (in secs) of display of fixation after curiosity rating |
| curiosity_tooSlow | 1 if time stamp response curiosity > response time window for curiosity rating - 3 * timing correction; else 0; subject sum score in other_information.csv ('curiosity_tooSlow') |
| mockOffset | time stamp (in secs) of offset of mock video |
| cueImage | time stamp (in secs) of cue image presentation |
| momentOfSurprise_1 | time stamp (in secs) of first moment of surprise |
| momentOfSurprise_2 | time stamp (in secs) of second moment of surprise |
| momentOfSurprise_3 | time stamp (in secs) of third moment of surprise |
| momentOfSurprise_4 | time stamp (in secs) of fourth moment of surprise |
| momentOfSurprise_5 | time stamp (in secs) of fifth moment of surprise |
| momentOfSurprise_6 | time stamp (in secs) of sixth moment of surprise |
| momentOfSurprise_7 | time stamp (in secs) of seventh moment of surprise |
| additionalMarker_momentOfSurprise_1 | time stamp (in secs) of first additional marker for moment of surprise |
| additionalMarker_momentOfSurprise_2 | time stamp (in secs) of second additional marker for moment of surprise |
| trialRecall | trial number in recall block of memory task |
| responseRecall | response given in cued recall block of memory task |
| cuedRecallStrict | dummy coding of cued recall performance according to strict criteria: 1 if recalled; else 0; subject sum score and percentage out of 36 trials in scores.csv ('cuedRecallStrict_abs and cuedRecallStrict_rel') |
| cuedRecallLenient | dummy coding of cued recall performance according to lenient criteria: 1 if recalled; else 0 subject sum score and percentage out of 36 trials in scores.csv ('cuedRecallLenient_abs and cuedRecallLenient_rel') |
| Flagging | 1 if cued recall response required further discussion during coding; else 0 |
| Comments | comments related to flagging |
| trialRecognition | trial number in recognition block of memory task |
| responseRecognition | response selected in recognition block of memory task |
| rtRecognition | response time for recognition response |
| responseConfidence | response given in confidence rating |
| rtConfidence | response time for response response |
| recognition | dummy coding of recogntion (regardless of confidence): 1 if correct answer chosen; else 0; subject sum score and percentage out of 36 trials in scores.csv ('allConf_abs and allConf_rel |
| recognitionConfLevel_4_5_6 | 1 if recognition = 1 & confidence > 3; else 0; subject sum score and percentage out of 36 trials in scores.csv ('highConf_abs and highConf_rel |
| rememberedStrictHigh | 1 if cuedRecallStrict = 1 or recognitionConfLevel_4_5_6 = 1; else 0; subject sum score and percentage out of 36 trials in scores.csv ('rememberedStrictHigh_abs and rememberedStrictHigh_rel |
| rememberedLenientHigh | 1 if cuedRecallLenient = 1 or recognitionConfLevel_4_5_6 = 1; else 0; subject sum score and percentage out of 36 trials in scores.csv ('rememberedLenientHigh_abs and rememberedLenientHigh_rel |

*Note*. Variable dictionary referencing the variables included in the MMC_experimental_data.csv file. An explanation of each variable is given to enhance usability for other researchers.

# References

Brainard, D. H. (1997). The Psychophysics Toolbox. *Spatial Vision*, *10*(4), 433–436. https://doi.org/10.1163/156856897X00357

Cacioppo, J. T., Petty, R. E., & Kao, C. F. (1984). The Efficient Assessment of Need for Cognition. In *Journal of Personality Assessment* (Vol. 48, Issue 3, pp. 306–307). https://doi.org/10.1207/s15327752jpa4803_13

Carver, C. S., & White, T. L. (1994). Behavioral Inhibition, Behavioral Activation, and Affective Responses to Impending Reward and Punishment: The BIS/BAS Scales. *Journal of Personality and Social Psychology*, *67*(2), 319–333. https://doi.org/10.1037/0022-3514.67.2.319

Elliot, A. J., & Harackiewicz, J. M. (1996). Approach and Avoidance Achievement Goals and Intrinsic Motivation: A Mediational Analysis. *Journal of Personality and Social Psychology*, *70*(3), 461–475. https://doi.org/10.1037/0022-3514.70.3.461

Elliot, A. J., & Thrash, T. M. (2010). Approach and Avoidance Temperament as Basic Dimensions of Personality. *Journal of Personality*, *78*(3), 865–906. https://doi.org/10.1111/j.1467-6494.2010.00636.x

Naylor, F. D. (1981). A State‐Trait Curiosity Inventory. *Australian Psychologist*, *16*(2), 172–183. https://doi.org/10.1080/00050068108255893

Pekrun, R., Goetz, T., Titz, W., & Perry, R. P. (2002). Academic emotions in students’ self-regulated learning and achievement: A program of qualitative and quantitative research. In *Educational Psychologist* (Vol. 37, Issue 2, pp. 91–105). Lawrence Erlbaum Associates Inc. https://doi.org/10.1207/S15326985EP3702_4

Ryan, R. M. (1982). Control and information in the intrapersonal sphere: An extension of cognitive evaluation theory. *Journal of Personality and Social Psychology*, *43*(3), 450–461. https://doi.org/10.1037/0022-3514.43.3.450

Spence, J. T., & Helmreich, R. L. (1983). Achievement-related motives and behavior. In J. T. Spence (Ed.), *Achievement and achievement motives: Psychological and socio-logical approaches* (pp. 10–74). W. H. Freeman.

Stoet, G. (2010). PsyToolkit: A software package for programming psychological experiments using Linux. *Behavior Research Methods*, *42*(4), 1096–1104. https://doi.org/10.3758/BRM.42.4.1096

Stoet, G. (2017). PsyToolkit: A Novel Web-Based Method for Running Online Questionnaires and Reaction-Time Experiments. *Teaching of Psychology*, *44*(1), 24–31. https://doi.org/10.1177/0098628316677643

Wigfield, A., & Eccles, J. S. (2000). Expectancy-value theory of achievement motivation. *Contemporary Educational Psychology*, *25*(1), 68–81. https://doi.org/10.1006/ceps.1999.1015

1. 50% additional bonus payment should have translated to £0.40 per correct answer. However, no participant reported to notice this error. [↑](#footnote-ref-1)
